# Synthesis and Pharmacological Characterization of Nociceptin/Orphanin FQ Dimeric Ligands

**DOI:** 10.1021/acs.jmedchem.5c02350

**Published:** 2025-11-04

**Authors:** Valentina Albanese, Pietro Pola, Michela Argentieri, Tiziano De Ventura, Alessia Frezza, Davide Illuminati, Davide Malfacini, Erika Marzola, Giulio Meneguzzo, Erika Morrone, Delia Preti, Alessandra Rizzo, Chiara Sturaro, Girolamo Calò, Remo Guerrini, Salvatore Pacifico, Chiara Ruzza

**Affiliations:** 1 Department of Chemical, Pharmaceutical and Agricultural Sciences, University of Ferrara, Via Luigi Borsari 46, 44121 Ferrara, Italy; 2 Department of Neuroscience and Rehabilitation, University of Ferrara, Via Luigi Borsari 46, 44121 Ferrara, Italy; 3 Department of Pharmaceutical and Pharmacological Sciences, Section of Pharmacology, University of Padova, Largo Meneghetti, 2, 35131 Padova, Italy; 4 Technopole of Ferrara, Laboratory for Advanced Therapies (LTTA), Via Fossato di Mortara 70, 44121 Ferrara, Italy

## Abstract

The neuropeptide nociceptin/orphanin FQ (N/OFQ) plays a key role in regulating several physiological functions and pathological states, which makes its receptor (NOP) a promising target for therapeutic interventions. In this study, we synthesized homodimeric N/OFQ-NH_2_ derivatives linked by disulfide bonds between cysteines appropriately introduced in the addressing region of the native peptide in place of the original amino acids. The *in vitro* activity of the compounds was evaluated using both an NOP-G protein interaction BRET assay and a calcium mobilization assay. The most potent compound, **1h** (pEC_50_ > 9), was obtained by coupling two monomeric precursors via a Leu^14^-to-Cys substitution. *In vivo*, **1h** demonstrated 3-fold greater potency than N/OFQ in eliciting loss of the righting reflex in mice and produced a long-lasting effect monitored for up to 7 h, supporting multimerization as a viable approach to developing long-acting peptide-based NOP ligands.

## Graphical abstract

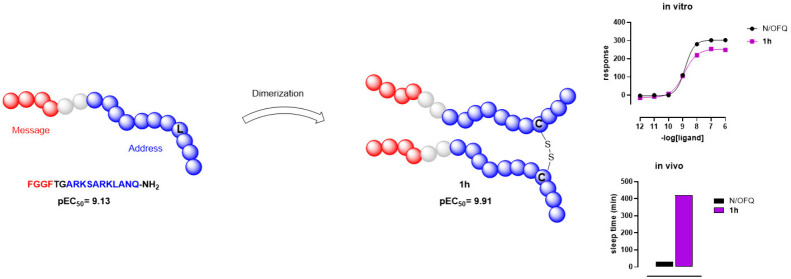

## Introduction

The nociceptin/orphanin FQ (N/OFQ) peptide receptor (NOP) is a class A G protein-coupled receptor (GPCR) that specifically binds the endogenous neuropeptide N/OFQ.^1,2^ Although classified within the opioid receptor family and sharing about 60% sequence homology with the classical opioid receptors (μ, δ, κ), the NOP receptor exhibits distinct pharmacological properties and ligand selectivity, reflecting unique features.^3^ N/OFQ is a heptadecapeptide (FGGFTG­ARKSAR­KLANQ) that combines a “message” domain (Phe^1^-Phe^4^), which is essential for receptor activation, and a C-terminal “address” region (Ala^7^-Gln^17^) that plays a key role in enhancing binding affinity and receptor selectivity.^4,5^ Initial X-ray crystallography studies^6,7^ provided the first structural insights into the inactive, antagonist-bound state identifying a ligand-binding pocket formed by transmembrane domains TM3, TM5, TM6, and TM7. Earlier work had already pointed to extracellular loop 2 (ECL2) as a critical element in receptor activation mechanisms.^8^ Recent cryo-EM studies have elucidated central structural features governing N/OFQ binding to the NOP receptor, revealing discrete conformational arrangements compared to antagonist-bound states.^9^ Upon activation, the NOP receptor couples to Gi/Go proteins, resulting in the inhibition of adenylate cyclase activity, activation of potassium conductance, and suppression of calcium channel function.^3^ N/OFQ and its receptors are extensively distributed across the central nervous system (CNS), with high expression levels in neuronal circuits and regions that play key roles in a variety of functions. These include pain modulation, learning and memory, emotional regulation, stress responses, reward processing and substance abuse, neuroendocrine regulation, appetite control, and motor functions.^3^ More recently, it has been demonstrated that the N/OFQ-NOP receptor system plays an important role in the control of the wake/sleep cycle.^10,11^ Given its broad physiological relevance, the N/OFQ-NOP receptor system represents a promising therapeutic target for various pathological conditions. Several lines of evidence demonstrated that peptide^4^ and nonpeptide^12^ NOP receptor agonists produce potent analgesic effects by intrathecal delivery in nonhuman primates.^13^ Moreover, mixed NOP/mu receptor agonists (i.e., cebranopadol) produce robust analgesic efficacy with reduced adverse effects typical of opioids.^14^ Of note, NOP receptor agonists were shown to be potentially useful in managing anxiety disorders,^15^ providing alternative options beyond current pharmacotherapies. In models of addiction, activation of the N/OFQ-NOP system has been shown to reduce drug-seeking behavior and attenuate withdrawal symptoms, highlighting its role in modulating reward pathways and stress responses.^16^ Furthermore, NOP agonists have been investigated as potential hypnotics.^11,17^

At present, only two small molecule NOP receptor agonists, namely, cebranopadol and sunobinop, are undergoing clinical evaluation. Cebranopadol is a first-in-class oral mixed agonist of the NOP and classical opioid receptors and has advanced to phase III clinical trials for the treatment of moderate-to-severe pain.^18,19^ Sunobinop, a NOP receptor selective partial agonist, is currently in phase II trials for insomnia related to alcohol cessation and alcohol use disorder.^11,20,21^

In addition to investigating non-peptide ligands for the NOP receptor, pharmaceutical research remains committed to developing stable and bioavailable peptide agonists, aiming to accelerate the translation of preclinical findings into innovative therapies. These efforts focus on optimizing the pharmacokinetic properties of the endogenous ligand while preserving its remarkable receptor affinity, selectivity, and efficacy. Herein, we describe our latest efforts in the development of N/OFQ oligomers derived from dimerization strategies of the native peptide. All compounds were evaluated *in vitro* in recombinant cells expressing the NOP receptor, using both a calcium mobilization assay performed on cells expressing the NOP receptor and chimeric G proteins^22^ and a bioluminescence resonance energy transfer (BRET) assay to assess NOP–G protein interaction.^23^ The most potent compound was further tested *ex vitro* in the electrically stimulated mouse vas deferens (mVD)^24^ and, considering the known pro-hypnotic effects of NOP receptor agonists,^11,17,25,26^ also evaluated *in vivo* for its ability to induce loss of the righting reflex (RR) in wild type and NOP knockout mice.

## Results

### Design and Synthesis of N/OFQ-Related Dimeric Peptides

In our previous work,^27^ we explored a series of homobivalent N/OFQ analogues by connecting the C-terminal of two N/OFQ(1–13)-NH_2_ fragments (the minimal active sequence) using various spacers. Building on these findings, we investigated a new dimerization approach using the full-length native sequence of N/OFQ-NH_2_. Specifically, we designed homodimer derivatives in which two N/OFQ-NH_2_ molecules are linked together through disulfide bonds between cysteine residues strategically introduced at different positions of the address domain (see the structures of compounds **1a**–**k** shown in Scheme 1).

**Scheme 1:**
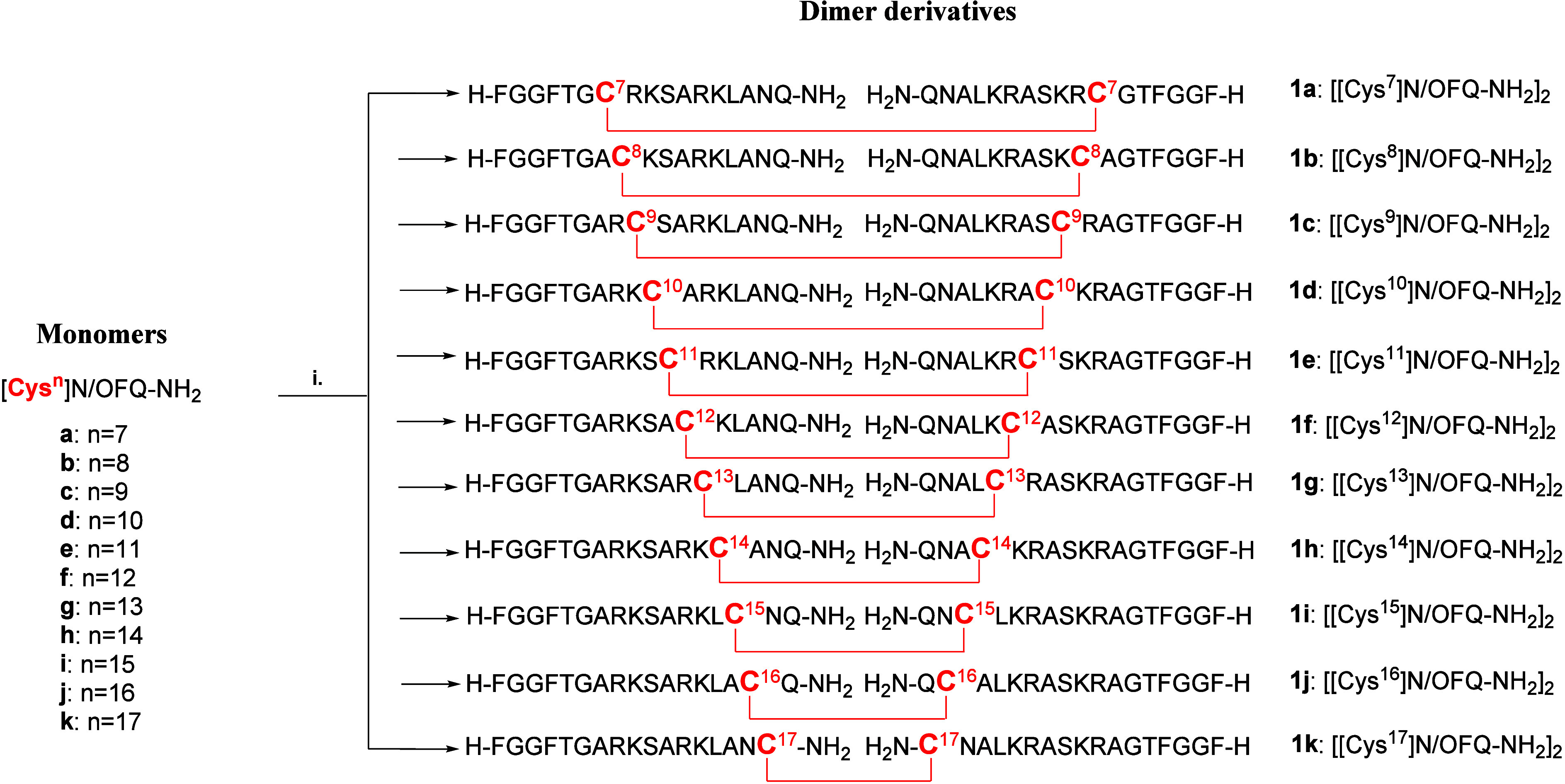
Synthesis of Homodimeric Peptides **1a**–**k** ^a^

a Reagents and conditions: (i) NaHCO_3_, H_2_O/CH_3_CN 1:1, 12 h, r.t.

The dimerization reaction involved air oxidation in the presence of a catalytic amount of NaHCO_3_ of the parent monomers ([Cys^*n*^]­N/OFQ-NH_2_ with *n* = 7 to 17) dissolved in a 50% v/v mixture of H_2_O and CH_3_CN.^27^ Each reaction was completed within 12 h, achieving full conversion of the monomers, simplifying the purification process in preparative HPLC. Dimers, after purification, showed a purity degree higher than 95% and were characterized by HRMS (HPLC and HRMS spectra reported in the Supporting Information).

### *In Vitro* Studies

The *in vitro* pharmacological activity of N/OFQ-NH_2_ and its novel dimeric derivatives was evaluated using both a NOP-G protein interaction BRET assay^23^ and a calcium mobilization assay performed in cells expressing the recombinant NOP receptor together with a chimeric G protein that force the coupling to Ca^2+^ release (Table 1 and Supporting Information Figures S1 and S2).^22^ N/OFQ-NH_2_ induced concentration-dependent NOP/G-protein interaction and calcium release, with potency and maximal effects superimposable to those of the natural peptide N/OFQ (Supporting Information Figures S1 and S2). N/OFQ exhibited potencies and maximal responses consistent with previously published data.^22,23^

**Table 1:** Pharmacological Activities of N/OFQ Dimeric Analogues^a^

		NOP–G protein interaction		Ca^2+^ mobilization	
		pEC_50_ (CL_95%_)	*E*_max_ ± sem	pEC_50_ (CL_95%_)	*E*_max_ ± sem
	N/OFQ-NH_2_	9.13 (8.67–9.60)	1.00	9.28 (9.02–9.54)	336 ± 16
**1a**	[[Cys^7^]N/OFQ-NH_2_]_2_	8.71 (8.13–9.28)	1.09 ± 0.09	7.57 (7.09–8.05)	293 ± 18
**1b**	[[Cys^8^]N/OFQ-NH_2_]_2_	8.04 (7.62–8.46)	1.04 ± 0.13	7.36 (6.42–8.30)	322 ± 20
**1c**	[[Cys^9^]N/OFQ-NH_2_]_2_	8.87 (8.36–9.38)	1.03 ± 0.13	8.30 (7.90–8.70)	289 ± 18
**1d**	[[Cys^10^]N/OFQ-NH_2_]_2_	9.85 (9.39–10.31)	1.07 ± 0.10	8.72 (8.28–9.17)	305 ± 42
**1e**	[[Cys^11^]N/OFQ-NH_2_]_2_	9.38 (8.85–9.92)	1.06 ± 0.09	8.49(7.61–9.37)	290 ± 42
**1f**	[[Cys^12^]N/OFQ-NH_2_]_2_	8.90 (8.45–9.34)	1.07 ± 0.12	8.52 (8.12–8.92)	346 ± 19
**1g**	[[Cys^13^]N/OFQ-NH_2_]_2_	9.52 (8.75–10.29)	0.84 ± 0.06	8.79 (8.18–9.40)	247 ± 39
**1h**	[[Cys^14^]N/OFQ-NH_2_]_2_	9.91 (9.14–10.68)	0.80 ± 0.08	9.14 (8.56–9.71)	241 ± 38
**1i**	[[Cys^15^]N/OFQ-NH_2_]_2_	9.71 (8.95–10.48)	0.95 ± 0.03	8.53(7.78–9.28)	297 ± 26
**1j**	[[Cys^16^]N/OFQ-NH_2_]_2_	9.76 (9.16–10.36)	0.98 ± 0.02	8.86 (8.35–9.37)	312 ± 38
**1k**	[[Cys^17^]N/OFQ-NH_2_]_2_	9.71 (8.99–10.43)	0.97 ± 0.03	8.52 (8.36–8.69)	317 ± 29

a pEC_50_ values are expressed as mean (CL_95%_); *E*_max_ values are expressed as mean ± sem; *N* = 5 experiments performed in duplicate.

Regarding the NOP-G protein interaction, all the synthesized compounds demonstrated remarkable potency, with EC_50_ values falling within the subnanomolar to low single-digit nanomolar range. The highest potency (pEC_50_ = 9.91) was achieved by connecting two monomeric precursors via a leucine-to-cysteine substitution at position 14 of the native sequence (compound **1h**, [[Cys^14^]­N/OFQ-NH_2_]_2_). A slight but significant reduction in potency occurs when the linkage between the two N/OFQ chains is positioned closer to the message domain (see compounds **1a**–**c**). A comparable trend was observed at position 12, where substituting the arginine residue (critical for target binding)^4^ with cysteine resulted in altered activity (compound **1f**).

In the calcium mobilization assay, the tested compounds confirmed nanomolar potency, albeit with slightly lower pEC_50_ values. Compound **1h** remained the most potent in the series, while a greater decrease in potency was observed when the branching is located near the message region. One-way ANOVA analysis revealed no statistically significant differences in *E*_max_ values among the compounds in either the NOP–G protein interaction or the calcium mobilization assay. Considering its promising profile *in vitro*, dimer **1h** was selected for further investigation.

Selectivity to the NOP receptor was carried out in cells expressing the μ, δ, and κ receptors and chimeric G proteins. Dermorphin, DPDPE ([d-Pen^2^,d-Pen^5^]­enkephalin), and dynorphin A produced a robust concentration-dependent stimulation of calcium release in cells expressing μ, δ, and κ receptors, respectively, with high maximal effects and potency values. Compound **1h** was always inactive up to 1 μM (Figure 1), demonstrating more than 1000-fold selectivity for the NOP over classical opioid receptors.

**Figure 1:**
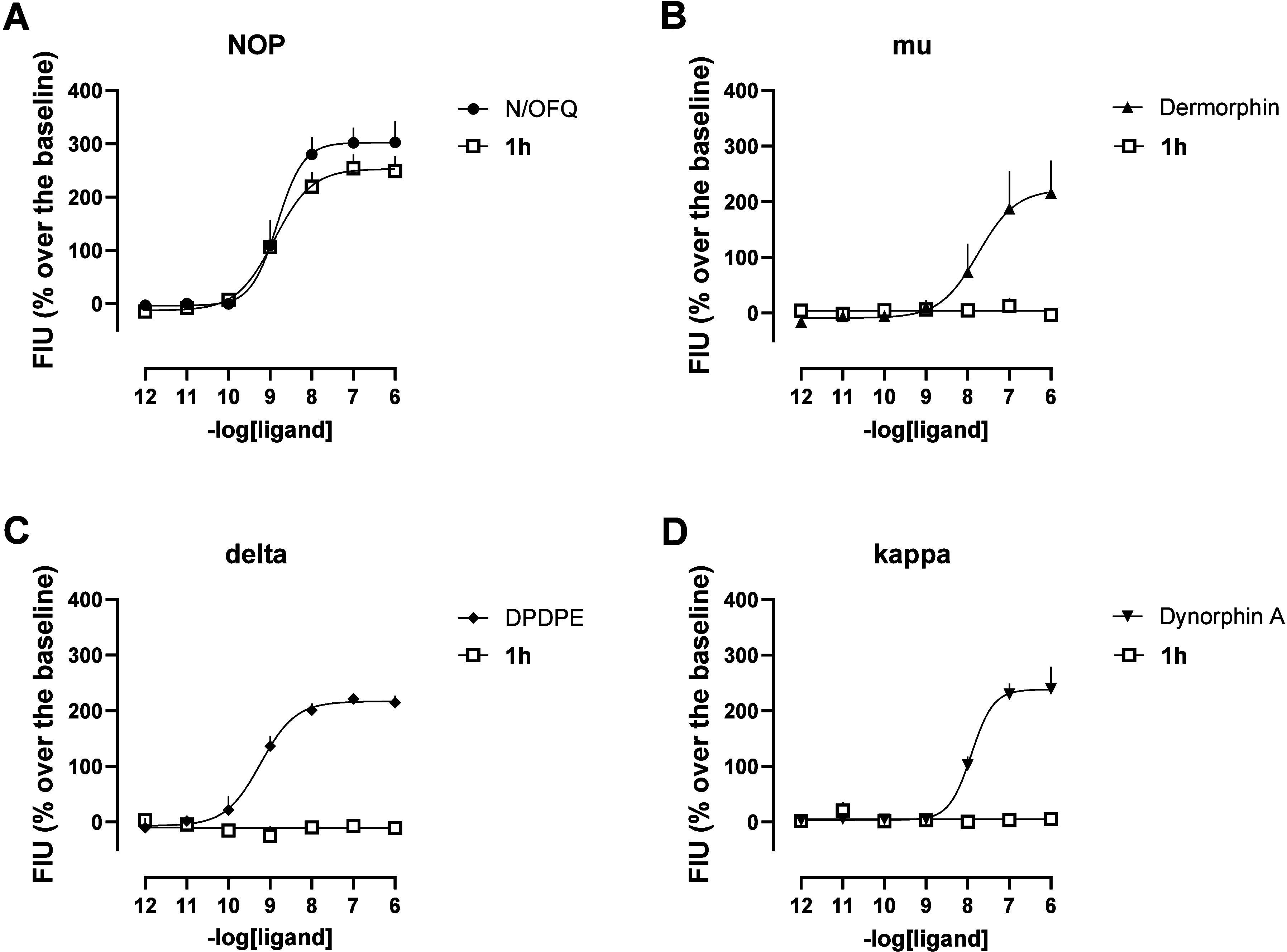
Compound **1h** selectivity of action at the NOP receptor in calcium mobilization experiments. Concentration–response curves to N/OFQ, dermorphin, DPDPE, and dynorphin A (i.e., standards) on CHO cells stably expressing the NOP (A), μ (B), δ (C), and κ (D) receptors and chimeric G proteins. Data are the mean ± sem of 5 experiments performed in duplicate.

In the electrically stimulated mVD bioassay, N/OFQ inhibited electrically induced contractions of tissues from wild-type mice (NOP­(+/+)) in a concentration-dependent manner with a potency of 7.59 (7.41–7.77) and a maximal inhibition of 71 ± 4%. A comparable inhibitory effect was observed with the delta receptor agonist DPDPE, which showed a potency of 7.72 (7.45–7.99) and a maximal effect of 86 ± 6%. Compound **1h** mimicked the inhibitory action of N/OFQ, producing a similar maximal effect but with lower potency (pEC_50_ = 6.79 (6.02–7.56)) (Figure 2A). No significant differences in the kinetics of action between N/OFQ and compound **1h** were observed; both peptides induced rapid inhibition of the electrically evoked twitch, which was fully and rapidly reversible upon washout. To investigate the receptor(s) mediating the action of compound **1h** in the mVD, experiments were conducted using tissues from NOP knockout mice (NOP­(−/−)). The concentration response curve to DPDPE was superimposable in tissues from both NOP­(+/+) and NOP­(−/−) mice. As expected, N/OFQ was completely inactive in tissues from NOP­(−/−) animals (Figure 2B). Similarly, compound **1h** showed no effect on NOP­(−/−) tissues.

**Figure 2:**
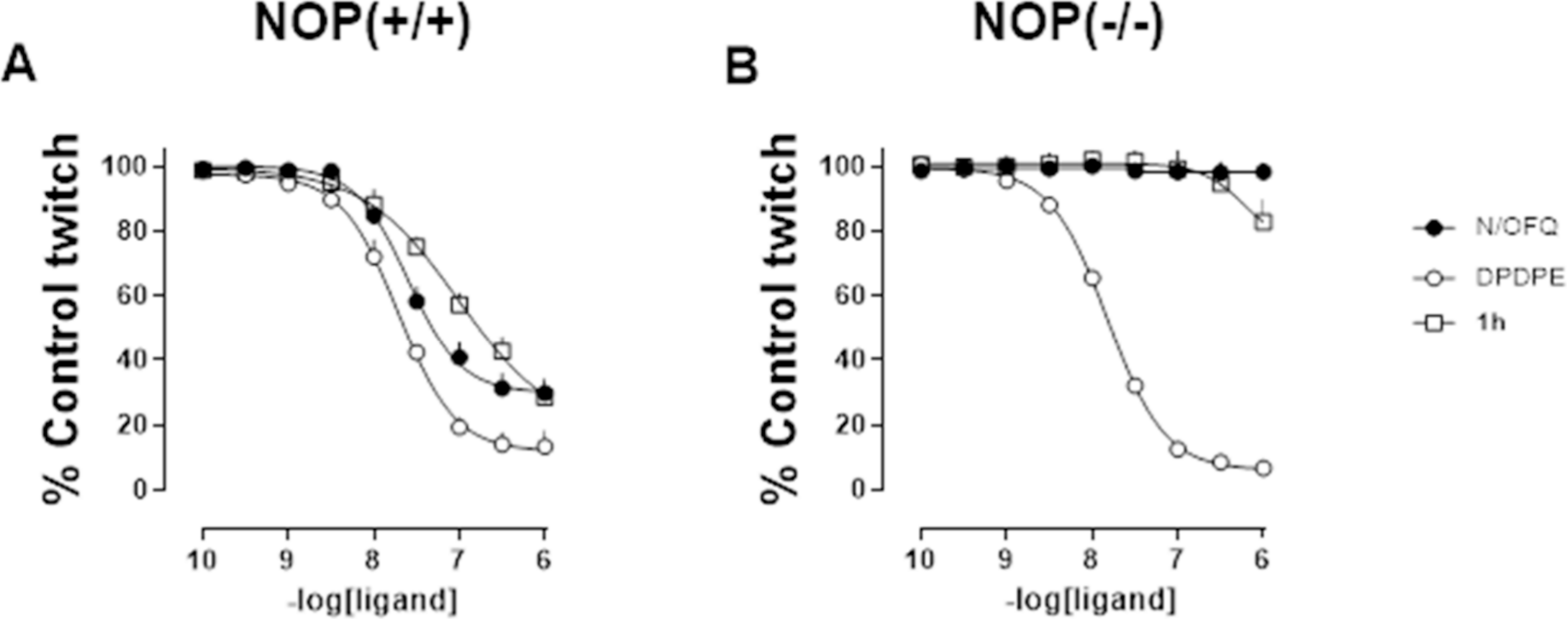
Mouse vas deferens bioassay. Concentration response curves to N/OFQ, DPDPE, and compound **1h** in NOP­(+/+) (A) and NOP­(−/−) (B) mice. Data are the mean ± sem of 5 experiments.

### *In Vivo* Studies

Since it has been reported that N/OFQ and NOP receptor agonists can induce loss of the righting reflex (RR) in mice,^25,26^ we evaluated the *in vivo* pharmacological activity of compound **1h** using this assay. The effects of **1h** were compared with those of its parent peptides N/OFQ and N/OFQ-NH_2_. In addition, we included in the study a dimeric derivative of N/OFQ(1–13)-NH_2_, previously reported by Pacifico et al. and referred to as compound **9** in the original publication,^27^ and the tetrabranched N/OFQ derivative PWT2-N/OFQ (see Supporting Information Figure S3).^42^ These compounds were tested in parallel with **1h** to investigate whether different dimerization/branching strategies may lead to distinct *in vivo* effects. At the high dose of 10 nmol, both N/OFQ and N/OFQ-NH_2_ induced loss of the RR in fewer than half of the treated mice, with a short-lasting action (Figure 3A–C). In contrast, compound **9** induced RR loss in 62% of mice at 3 nmol and in all of the animals at 10 nmol (Figure 3D). In both cases, mice that lost the RR remained asleep until the experimental cutoff of 420 min (Figure 3F). PWT2-N/OFQ produced similar effects but was active starting from the dose of 0.3 nmol, which caused RR loss in 50% of treated mice (Figures 3G [Fig fig3]I). Results obtained with compound **1h** were similar to those of compound **9**; in fact, 75% of mice treated with 3 nmol and all mice treated with 10 nmol lost the RR, with a similarly prolonged effect lasting up to the 420 min cutoff (Figure 3L–N). These results suggest that N/OFQ dimerization enhances both *in vivo* potency and, more markedly, the duration of action, resulting in potent and long-lasting peptide agonists. However, no significant differences in *in vivo* activity were observed between the two dimerization strategies, leading to compound **9** and compound **1h**. The branching strategy used to obtain PWT2-N/OFQ appeared to confer slightly greater potency compared to the dimers, although no major differences were observed in terms of the duration of action. PWT2-N/OFQ was the only compound showing a significantly longer latency to RR loss compared to both N/OFQ and N/OFQ-NH_2_. In contrast, compounds **9** and **1h** required a similar time from administration to RR loss, indicating that the enhanced duration of action observed with these dimers is not associated with delayed onset.

**Figure 3:**
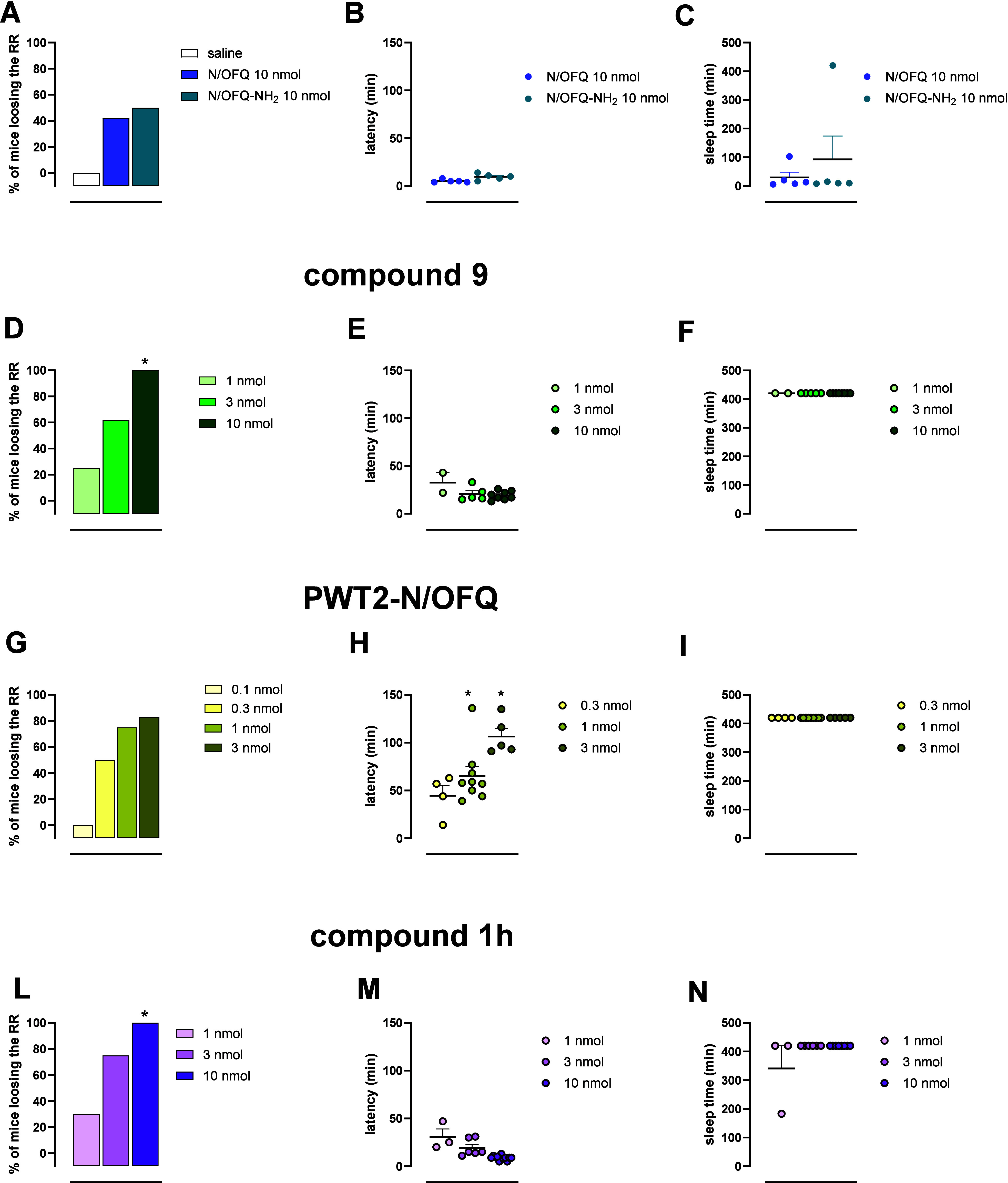
Loss of the RR assay. Panels A–C: N/OFQ and N/OFQ-NH_2_ (10 nmol, icv). Panels D–F: compound **9** ^27^ (1–10 nmol, icv). Panels G–I: PWT2-N/OFQ^42^ (0.1–3 nmol, icv). Panels L–N: compound **1h** (1–10 nmol, icv). Left panels show the percentage of animals that lost the RR; middle panels show the latency to lose the RR from compound injection; right panels show sleep duration. Sleep duration is defined as the interval between loss and recovery of the RR. Data are expressed as a percentage (RR loss) or mean and sem (latency and sleep duration). *N* = 8–10 mice per group; sleep-duration and latency graphs include only those animals that lost the RR. % of mice losing the RR: *p < 0.05 vs N/OFQ-NH_2_, according to Fisher’s exact test. Latency: *p < 0.05 vs N/OFQ-NH2, according to one-way ANOVA followed by Dunnet’s post hoc test (*F*(7,47) = 39.61). Groups with *N* less than 5 were excluded from the analysis.

Finally, the selectivity of action of **1h** has been tested *in vivo* by treating NOP­(−/−) mice with 10 nmol of the compound. Compound **1h** resulted completely inactive in NOP­(−/−) mice (Figure 4A,B). On the contrary, diazepam (15 mg/kg, ip) produced similar hypnotic effects in NOP­(+/+) and NOP­(−/−) mice, suggesting a similar sensitivity of the two genotypes to classical hypnotic drugs (Figure 4C,D).

**Figure 4:**
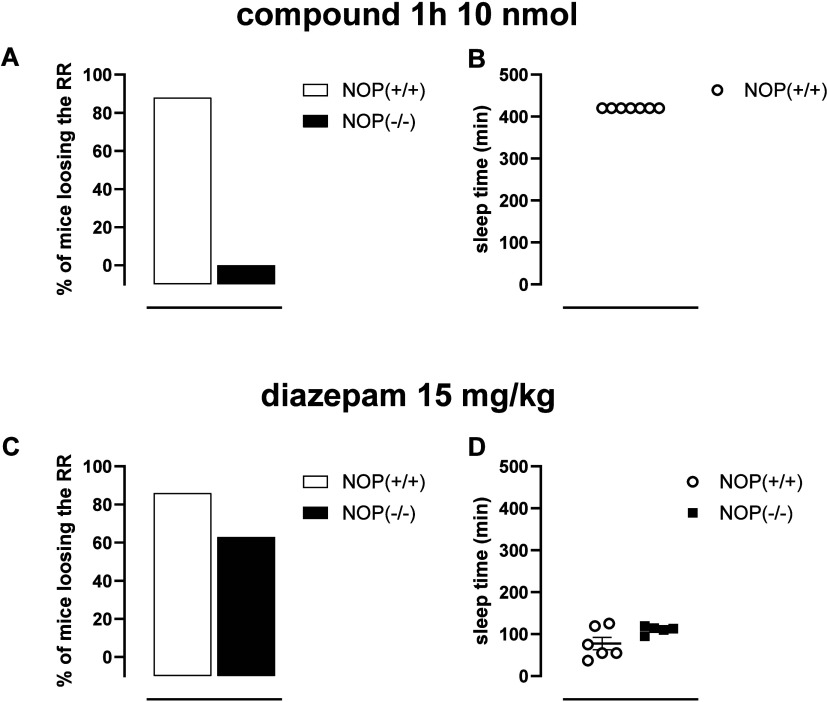
Loss of the RR assay. (A, B) Compound **1h** (10 nmol, icv) in NOP­(+/+) and NOP­(−/−) mice. (C, D) Diazepam (15 mg/kg, ip) in NOP­(+/+) and NOP­(−/−) mice. Left panels show the percentage of animals that lost the RR; right panels show their sleep duration. Sleep duration is defined as the interval between the loss and recovery of the RR. Data are expressed as a percentage (RR loss) or mean ± sem (sleep duration). *N* = 7–9 mice per group; sleep-duration graphs include only those animals that lost the RR.

## Discussion

To overcome the typical limitations of therapeutic peptides, including poor stability *in vivo*, rapid enzymatic degradation, limited bioavailability, and difficulty in reaching target sites effectively, various approaches have been explored. Among these, peptide oligomerization has emerged as a particularly promising strategy aimed at enhancing the pharmacokinetic properties. For example, homobivalent peptide derivatives have, in certain cases, shown a notable improvement in pharmacological properties over their monomeric counterparts, resulting in GPCR ligands with increased potency both *in vitro* and *in vivo*.^28–30^ The enhanced biological activity seen in dimeric peptides is partly attributed to their capacity for multivalent interactions, allowing them to simultaneously bind multiple receptor sites. Over the past 40 years, extensive research on both peptide and nonpeptide multivalent ligands has provided strong evidence that many GPCRs exist not only as monomers but also as higher-order oligomers, particularly tetramers.^31^ Multimeric constructs can indeed facilitate targeting of complex receptor systems, leading to more precise modulation of physiological responses and fine-tuning of receptor signaling pathways. Moreover, the greater steric hindrance resulting from dimerization can improve resistance to enzymatic degradation, thereby enhancing peptide stability and extending their half-life *in vivo*.^32–35^ This may lead to clinical advantages such as longer lasting action and consequently less frequent dosing.

In the past, the multimerization approach has been explored to develop N/OFQ-related peptides with enhanced pharmacological properties. Particularly, the C-terminal portion of N/OFQ has been exploited for generating homo^27^ and hetero^36–38^ bivalent NOP ligands as well as homo^39^ and hetero^40^ tetrameric NOP ligands. While multiple lines of evidence have been gathered indicating that tetramerization of N/OFQ positively influences their half-life when administered *in vivo*,^4,39,41–43^ the impact of dimerization on the duration of action of bioactive peptides remains less thoroughly investigated.

To these purposes, we recently investigated a series of homobivalent N/OFQ analogues developed by linking the C-terminal of two N/OFQ(1–13)-NH_2_ units (as the minimal active sequence) via various spacers.^27^ The study showed that *in vitro* activity remained mostly unchanged by ligand dimerization or variations in the spacer’s length and composition. However, dimerization of the low-potency analogue N/OFQ(1–12)-NH_2_ resulted in a complete restoration of potency in a mouse vas deferens assay. Similarly, when N/OFQ(1–12)-NH_2_ was conjugated with the biologically inactive N/OFQ(2–12)-NH_2_ (lacking the N-terminal F residue), the resulting heterodimer exhibited a potency comparable to that of N/OFQ(1–13)-NH_2_.

These data suggested that dimerization at the address level, rather than at the message domain, is a key factor for the recognition and activation of the NOP receptor by the dimer peptide. The assumption prompted us to synthetise and investigate, both *in vitro* and *in vivo*, compounds **1a**–**k** described in this work and obtained through new dimerization strategies focused on the address domain.

For the *in vitro* characterization of all of the compounds, the G protein and calcium mobilization assays were used. These assays, differing in signal amplification, provide complementary information, allowing a more accurate evaluation of compound efficacy, which can vary depending on the system employed.^44^ All of the synthesized compounds behaved as potent full agonists of the NOP receptor. The most potent compound was obtained by linking two monomeric precursors via cysteine residues at the 14-position (compound **1h**, [[Cys^14^]­N/OFQ-NH_2_]_2_). A slight loss in potency was observed when the branching was positioned close to the massage domain (see compounds **1a**–**c**) or when it required the replacement of amino acids important for target binding such as the positively charged Arg^12^ (compound **1f**).^4^ In the case of **1h**, the insertion of a cysteine at position 14 replaces a leucine residue in the native sequence nonessential for biological activity.^45^ Given its high potency, the pharmacological profile of compound **1h** was further investigated in the electrically stimulated mVD assay, a well-known N/OFQ sensitive pharmacological preparation, that offers the opportunity to characterize the pharmacological activity of NOP ligands at the murine receptor in its native environment.^24,46^ Compound **1h** acted as a full NOP agonist, slightly less potent than N/OFQ and exhibited a rapid onset of action and full reversibility upon washout, mimicking the kinetic behavior of the natural ligand. Comparison with previously reported N/OFQ dimeric/branched derivatives, such as compound **9** ^27^ and the tetrabranched PWT2-N/OFQ,^42^ reveals some differences. While compound **9** and PWT2-N/OFQ showed a modest increase in potency, **1h** displayed a ∼3-fold decrease compared to that of N/OFQ. Although we cannot currently explain this divergence, these variations are relatively minor, supporting the general observation that dimerization or branching has only a limited impact on NOP receptor activation *in vitro*. More notable are the kinetic differences. PWT2-N/OFQ showed a slow and poorly reversible interaction with the receptor,^42^ suggesting that extensive branching may interfere with binding and dissociation kinetics. In contrast, both compound **9** ^27^ and **1h** maintained rapid and reversible kinetics, implying that dimerization does not significantly affect the binding dynamics. Finally, we assessed the selectivity of compound **1h** through calcium mobilization assays in cells expressing recombinant μ, δ, and κ opioid receptors as well as in mVD preparations from NOP­(−/−) mice. In both systems, **1h** was inactive at the concentrations tested, demonstrating high selectivity for the NOP receptor. This contrasts with PWT2-N/OFQ, which, although inactive in recombinant cell lines, elicited weak effects in NOP­(−/−) tissues at high concentrations, suggesting some loss of selectivity.^42^ Overall, compound **1h** shows an improved selectivity profile relative to PWT2-N/OFQ. Encouraged by these results, **1h** was examined for its potential to induce a loss of the RR in mice. Compound **9** was included in the study as an example of a linear dimeric derivative with *in vitro* potency comparable to that of **1h**, albeit synthesized through a different fusion approach.^27^ The aim was to compare the *in vivo* profile of a branched peptide dimer (**1h**) with that of a linear congener (**9**). The tetrameric derivative PWT2-N/OFQ has been included in the study to test whether tetramerization may offer an advantage over dimerization. While the pharmacological activity of compound **9** has so far been characterized only *in vitro*, PWT2-N/OFQ has been extensively studied *in vivo* across various assays and always in comparison with N/OFQ. These comprehensive *in vivo* studies consistently demonstrated that PWT2-N/OFQ mimicked the actions of the natural peptide N/OFQ but was more potent (approximately 30-fold^41–43^ ), elicited larger effects (e.g., ref 42), and exhibited a remarkably prolonged duration of action.^43^ Of note, no *in vivo* selectivity issues were detected for PWT2-N/OFQ. Both NOP­(−/−) and antagonism studies robustly demonstrated that its effects are solely due to selective NOP receptor activation.^41,43^ Here compound **1h** resulted 3-fold more potent than N/OFQ and N/OFQ-NH_2_ in inducing loss of the RR reflex in mice. Moreover, while in those mice that lost the RR N/OFQ and N/OFQ-NH_2_ effects lasted only few minutes, **1h** produced a long-lasting effect that reached the experimental cutoff of 7 h. Importantly, **1h** was completely inactive in NOP­(−/−) mice, demonstrating that the mechanism by which it induces the loss of RR is the selective activation of the NOP receptor. Of note, no differences in the sensitivity to the hypnotic effects of diazepam were measured between NOP­(+/+) and NOP­(−/−) mice, suggesting that the inactivity of **1h** in NOP­(−/−) mice is not due to a reduced sensitivity to hypnotic substances of the mutant mice. Compound **9** displayed a similar *in vivo* activity to **1h**, in terms of both potency and duration of action. Thus, both dimerization approaches were useful to obtain more potent and longer-lasting peptides, with no significant advantages of one strategy over the other one. The tetrabranched peptide proved somewhat advantageous in terms of potency, being 10-fold more potent than compounds **9** and **1h** and, in line with previous studies,^41–43^ 30 times more potent than N/OFQ. However, the duration of action was similarly high for both dimeric and tetra-branched derivatives (at least 40 times longer than that of N/OFQ), suggesting that neither multimerization strategy offers a clear superiority over the other. However, since all compounds reached the assay cutoff for duration of action, it is not possible to draw definitive comparisons between the three compound types. Regarding the onset of action, no statistically significant differences were observed among N/OFQ, N/OFQ-NH_2_, compound **9**, and compound **1h**, all showing a rapid induction of the effect. In contrast, PWT2-N/OFQ required a longer time to induce the loss of the RR, a slow onset that is consistent with previous reports in the literature.^42^ Thus, although tetramerization appears to be associated with a delayed onset of action, this was not observed with dimeric derivatives. What emerges from the *in vivo* results of this study is that icv administration of peptides with a longer duration of action compared to the very short-acting N/OFQ leads to a remarkably prolonged loss of the RR reflex in mice. This effect appears to last longer than what has been reported in the literature for nonpeptide NOP agonists such as Ro 64-6198 ^17^ or Sunobinop,^11^ which, at EEG recordings, induced sleep for approximately 3 to 5 h in rats. Although the methodologies differ and a direct comparison is not possible, the strikingly long-lasting RR suppression observed here remains difficult to explain. One possible explanation is that the central route of administration used in this study allows the peptides to reach very high local concentrations in specific brain regions, which may be less accessible when the compounds are administered systemically. However, this remains a working hypothesis, and further neurobiological investigations are needed to better define the mechanisms and neural circuits underlying the NOP agonist-induced sleep. Overall, what can be concluded from this assay is that all of the multimeric peptides tested here appeared to be long-acting. Future studies using alternative behavioral assays will be necessary to further clarify the pharmacokinetic properties and duration of action of these compounds *in vivo*.

## Conclusions

In this study, we reported the discovery and characterization of novel peptide ligands targeting the NOP receptor, designed through a dimerization strategy of N/OFQ that led to the branching of the peptide address domain. The approach adopted provided the analogue **1h** that was 3-fold more potent than N/OFQ in inducing the loss of the RR reflex in mice, with a long-lasting effect monitored for 7 h. The comparison with the dimeric N/OFQ analogue compound **9** revealed that peptide dimerization, independent from the final architecture (linear or branched), is responsible for the significant increase in the *in vivo* duration of action. Contrary to peptide tetramerization as in PWT2-N/OFQ, dimerization does not significantly affect the NOP binding dynamics. Our findings contribute to the expanding repertoire of NOP receptor agonists and provide insights into the structural and functional advantages conferred by peptide dimerization, advancing the development of therapeutics targeting N/OFQ pathways.

## Materials and Methods

### Solid Phase Peptide Synthesis

All monomers were synthesized via solid-phase peptide synthesis (SPPS) by using standard Fmoc/tBu chemistry. Fmoc-amino acids were purchased from BLDpharm, Zentek, and Sigma-Aldrich. Polystyrene resin functionalized with a Rink Amide AmphiSpheres 20 RAM linker (derivatization: 0.55 mmol/g; 75–150 μm) served as the solid support, enabling C-terminal amidation upon cleavage. Synthesis was performed on a SyroXP automatic peptide synthesizer. The protocol involved sequential cycles of coupling, capping, and Fmoc deprotection. Fixed concentrations of HBTU (0.62 M), DIPEA (0.87 M), Ac_2_O (0.5 M), NMM (0.25 M), and piperidine (40%) in DMF were used for automated reaction cycles. Peptide cleavage from the solid support was achieved using a cleavage cocktail composed of TFA/triisopropylsilane/H_2_O (95:2.5:2.5) at room temperature for 4 h (10 mL). The resin was removed by filtration, and the filtrate was first concentrated by rotary evaporation and then treated with Et_2_O at −20 °C to precipitate the peptides. Finally, the precipitated peptides were centrifuged and dried. Crude reaction products were purified via a reverse-phase 1260 Infinity II preparative LC system equipped with a Jupiter (Phenomenex) C18, 15 μm, 300 Å column (250 mm × 30 mm). Elution was performed with a linear gradient of solvents A (100% H_2_O and 0.1% TFA) and B (40% H_2_O, 60% CH_3_CN, and 0.1% TFA) at a flow rate of 20 mL/min. The gradient program was optimized based on the analytical HPLC profile of the crude peptide. The molecular weights of reaction intermediates and final products were determined by using an ESI (electrospray ionization) MICROMASS ZMD 2000 mass spectrometer. The purity of the final compounds was determined by analytical reverse-phase HPLC on an Agilent Technologies 1200 series system equipped with a UV detector. A Kinetex (Phenomenex) 5 μm C18 100 Å (150 mm × 4.6 mm) LC column was used, eluted with a polar mobile phase consisting of solvent A (100% H_2_O, 0.1% TFA) and solvent B (100% CH_3_CN, 0.1% TFA). A linear gradient from 100% A to 100% B was applied over 25 min. All final compounds were >95% pure by HPLC analysis (see Supporting Information for HPLC profiles). High resolution masses for the final compounds were determined on a Vanquish Flex UHPLC coupled to an Orbitrap Exploris 120 HRMS instrument (see Supporting Information).

### General Procedure for the Synthesis of the Dimeric Peptides

To a solution of each monomer ([Cys^*n*^]­N/OFQ-NH_2_, 10 μmol) in a mixture of H_2_O/CH_3_CN (1:1, 1 mL), 5 μL of a 5% aqueous NaHCO_3_ was added. The reaction progress was monitored via ESI-MS. After it was completed, the crude mixture was purified by preparative HPLC.

### Calcium Mobilization Assay

Chinese hamster ovary (CHO) cells stably coexpressing the human NOP, μ, or κ opioid receptors and the Gα_qi5_ protein or the human δ and the Gα_qG66Di5_ protein were used in this assay.^22,47^ Cells were cultured in DMEM/F-12 (1:1) medium supplemented with 10% FBS, 2 mM l-glutamine, 200 mg/mL G418, 100 mg/mL hygromycin B, 100 IU/mL penicillin, and 100 IU/mL streptomycin. Cells were maintained at 37 °C in a humidified atmosphere with 5% CO_2_ and were seeded at 50 000 cells/well into 96-well black, clear-bottom plates 24 h before test. Loading for 45 min with a solution consisting of HBSS supplemented with 2.5 mM probenecid, 3 μM Fluo-4 AM, and 0.01% pluronic acid ensures the calcium-sensitive dye (Fluo-4) reaches the needed concentration inside the cell, while the exchange of the solution with 100 μL/well of buffer consisting of HBSS with 20 mM HEPES, 2.5 mM probenecid, and 500 μM Brilliant Black allows for fluorescence background to decrease. Serial dilutions of ligands were prepared in HBSS buffer with 20 mM HEPES and 0.02% bovine serum albumin (BSA) to minimize the ligands’ stickiness to plasticware. The automated microplate reader FlexStation II (Molecular Devices, CA, US) was employed at 37 °C to detect changes in fluorescence intensity. The effects of all compounds were expressed as the maximum change in percentage over the baseline fluorescence measured in samples treated with vehicles.

### NOP Receptor–G Protein Interaction

A BRET interaction assay was used, as previously detailed in ref 23, to study the propensity of ligands to evoke interaction between the NOP receptor and to G protein. Membranes taken from HEK293 cells stably coexpressing the fusoproteins NOP-RLuc and Gβ1-RGFP were used. Such cells were grown in DMEM medium supplemented with 10% FBS, 2 mM l-glutamine, 200 mg/mL G418, 100 mg/mL hygromycin B, 100 IU/mL penicillin, and 100 IU/mL streptomycin in a humidified atmosphere with 5% CO_2_ at 37 °C. Cell membranes were thawed and resuspended in PBS supplemented with 0.01% BSA before the assay, and an amount of 3 μg of total protein was dispensed in each of the 96 wells together with 2 μM Prolume Purple Coelenterazine. All experiments were carried out at room temperature. Ligands were added, and averaged BRET ratios from 10 min measurements were computed. BRET ratios were computed as counts per second (CPS) obtained on a Victor Nivo (PerkinElmer) with 405 (10) nm and 510 (30) nm bandpass filters. Vehicles’ BRET ratios were derived from light passed through 510 divided by that passed through 405 filters. Vehicles BRET values were subtracted from all computations, and data sets were normalized as a fraction of N/OFQ maximal effects.

### Mouse Vas Deferens Bioassay

This study was approved by the Animal Welfare Body of the University of Ferrara and by the Italian Ministry of Health (authorization number CBCC2.N.BXI). Experiments were performed on an isolated mouse vas deferens. All experiments were conducted with mice bred and housed in the University of Ferrara’s animal facility under specific pathogen-free conditions (SPF). All mice were housed in cages with individual ventilation, with a constant temperature of 21 °C, 60% humidity, and a 12 h light/dark cycle. Food and water were provided ad libitum. CD-1 NOP­(+/+) and CD-1 NOP­(−/−) male mice, aged 9 to 12 months, were used. Animals were sacrificed on the day of the experiment with CO_2_ overdose. Bioassay experiments were performed as previously described.^24^ The tissues were suspended in 5 mL organ bath containing Krebs solution (NaCl 118.5 nM, KCl 4.7 nM, KH_2_PO_4_ 1.2 nM, NaHCO_3_ 25 nM, CaCl_2_ 2.5 nM, glucose 10 nM). The Krebs solution was oxygenated with 95% O_2_ and 5% CO_2_ and the temperature set at 33 °C with a resting tension of 0.3 g applied to the tissues. Tissues were stimulated through two platinum electrodes with a supramaximal rectangular pulse of 1 ms duration, 0.05 Hz frequency, and 80 V of amplitude. The electrically evoked contractions were measured isotonically by means of Basile strain gauge transducers (Basile, IT) and a recorder with a Power Lab 8 instrument (ADInstruments, CO, U.S.). Following an equilibration period of approximately 60 min, the contractions induced by electrical field stimulation were stable. At this time, cumulative concentration response curves were carried out.

### Loss of the Righting Reflex Assay

All of the experimental procedures adopted in the *in vivo* studies comply with the European Directive 2010/63/EU on protecting animals used for scientific purposes and Italian Legislative Decree no. 26 of March 4, 2014. These experiments were approved by the Animal Welfare Body of the University of Ferrara and by the Ministry of Health (authorization number 677/2024-PR). *In vivo* studies have been reported following ARRIVE guidelines.^48^ All experiments were conducted with mice bred and housed in the University of Ferrara’s animal facility under specific pathogen-free conditions. All mice were housed in cages with individual ventilation, with a constant temperature of 21 °C, 60% humidity, and a 12 h light/dark cycle. Food and water were provided ad libitum. CD-1 male and female mice, aged 2 to 4 months, were used. Details about the generation of NOP­(−/−) and NOP­(+/+) mice have been published previously;^49,50^ these mice have been backcrossed on CD-1 strain in our laboratories. NOP­(+/+) and NOP­(−/−) littermates were obtained by mating with NOP(±) mice. All mice were genotyped using the polymerase chain reaction previously described.^51^ All mice were used only once. The RR assay was performed as previously described.^52^ Mice were given an intracerebroventricular (icv) injection of saline, N/OFQ (10 nmol), N/OFQ-NH_2_ (10 nmol), compound **9** (1–10 nmol),^27^ PWT2-N/OFQ (0.1–3 nmol),^42^ or compound **1h** (1–10 nmol). Diazepam 15 mg/kg was injected ip. When the animals lost RR, they were placed in a plastic cage, and the time was recorded by an expert observer, blind to drug treatments and/or genotype. Animals were judged to have regained the RR response when they could correct themselves three times within 30 s. Sleeping time is defined as the amount of time between the loss and regaining of the RR; it was rounded to the nearest minute.

### Data Analysis, Statistics, and Terminology

Concentration-response curves to agonists were analyzed by a four-parameter logistic nonlinear regression model: Effect = baseline + (*E*_max_ – baseline)/(1 + 10^((logEC_50_–log[ligand])slope)^). In BRET the effects were normalized to that of N/OFQ-NH_2_ (*E*_max_ = 1). Experimental data were expressed as mean ± sem of at least 5 experiments. Potency values were expressed as the mean and CL_95%_. *In vitro* *E*_max_ values were analyzed by one-way ANOVA, while *in vivo* latency data were evaluated by one-way ANOVA followed by Dunnett’s post hoc test. The proportion of mice losing the righting reflex was compared by Fisher’s exact test performed on raw data. GraphPad Prism 10.0 software was used for all of the analyses. The terminology employed is consistent with the International Union of Basic and Clinical Pharmacology (IUPHAR) recommendations.^53,54^

## Supplementary Material





## Notes

Figures S1 and S2 (NOP–G protein interaction assay and calcium mobilization assay); Figure S3 (chemical structure of compound **9** and PWT2-N/OFQ); HRMS spectra (pp S6–S16) and HPLC traces (pp S17–S22) of dimeric peptides **1a**–**k** (PDF)
Molecular formula strings and some data (CSV)

## Abbreviations Used

BRET: bioluminescence resonance energy transfer
BSA: bovine serum albumin
CHO cells: Chinese hamster ovary cells
CL95%: 95% confidence limit
DIPEA: *N*,*N*-diisopropylethylamine
FBS: fetal bovine serum
HBSS: Hanks’ balanced salt solution
HBTU: *N*,*N*,*N*′,*N*′-tetramethyl-*O*-(1*H*-benzotriazol-1-yl)­uronium hexafluorophosphate
mVD: mouse vas deferens
N/OFQ: nociceptin/orphanin FQ
NOP: nociceptin/orphanin FQ (N/OFQ) peptide receptor
PWT: peptide welding technology
RR: loss of the righting reflex
sem: standard error of the mean
SPPS: solid-phase peptide synthesis
