# Supplementary material for: Synthesis and Pharmacological Characterization of Nociceptin/Orphanin FQ Dimeric Ligands
Source: J Med Chem. 2025 Nov 4;68(21):23448–58. doi: 10.1021/acs.jmedchem.5c02350 (PMC12621184; doi:10.1021/acs.jmedchem.5c02350)
Supplement: Supplementary file 1 [file jm5c02350_si_001.pdf]

Supporting Information for

Synthesis and pharmacological characterization of nociceptin/orphanin FQ dimeric ligands.

Valentina Albanese[1#], Pietro Pola [2#], Michela Argentieri[2], Tiziano De Ventura[1], Alessia Frezza[2], Davide Illuminati[1], Davide Malfacini[3], Erika Marzola[1], Giulio Meneguzzo[1], Erika Morrone[3], Delia Preti[1], Alessandra Rizzo[1], Chiara Sturaro[2], Girolamo Calò[3], Remo Guerrini[1,4], Salvatore Pacifico[1*], Chiara Ruzza[2].

[1]*Department of Chemical, Pharmaceutical and Agricultural Sciences, University of Ferrara, Via Luigi Borsari 46, 44121 Ferrara, Italy.*

[2]*Department of Neuroscience and Rehabilitation, University of Ferrara, Via Luigi Borsari 46, 44121 Ferrara, Italy.*

[3]*Department of Pharmaceutical and Pharmacological Sciences, Section of Pharmacology, University of Padova, Largo Meneghetti, 2, 35131 Padova, Italy.*

[4]*Technopole of Ferrara, Laboratory for Advanced Therapies (LTTA), via Fossato di Mortara 70, 44121 Ferrara, Italy*

*Corresponding author:
Salvatore Pacifico, e-mail: pcfsvt@unife.it

**CONTENTS**	**Pag.**
Figure S1. NOP – G protein interaction assay	*S2*
Figure S2. Calcium mobilization assay	*S3*
Figure S3. Chemical structure of compound 9 and PWT2-N/OFQ	*S4*
HRMS method	*S5*
HRMS spectra and isotopic composition of dimeric peptides **1a-k**	*S6-16*
HPLC traces of the final dimeric peptides **1a-k**	*S17-22*

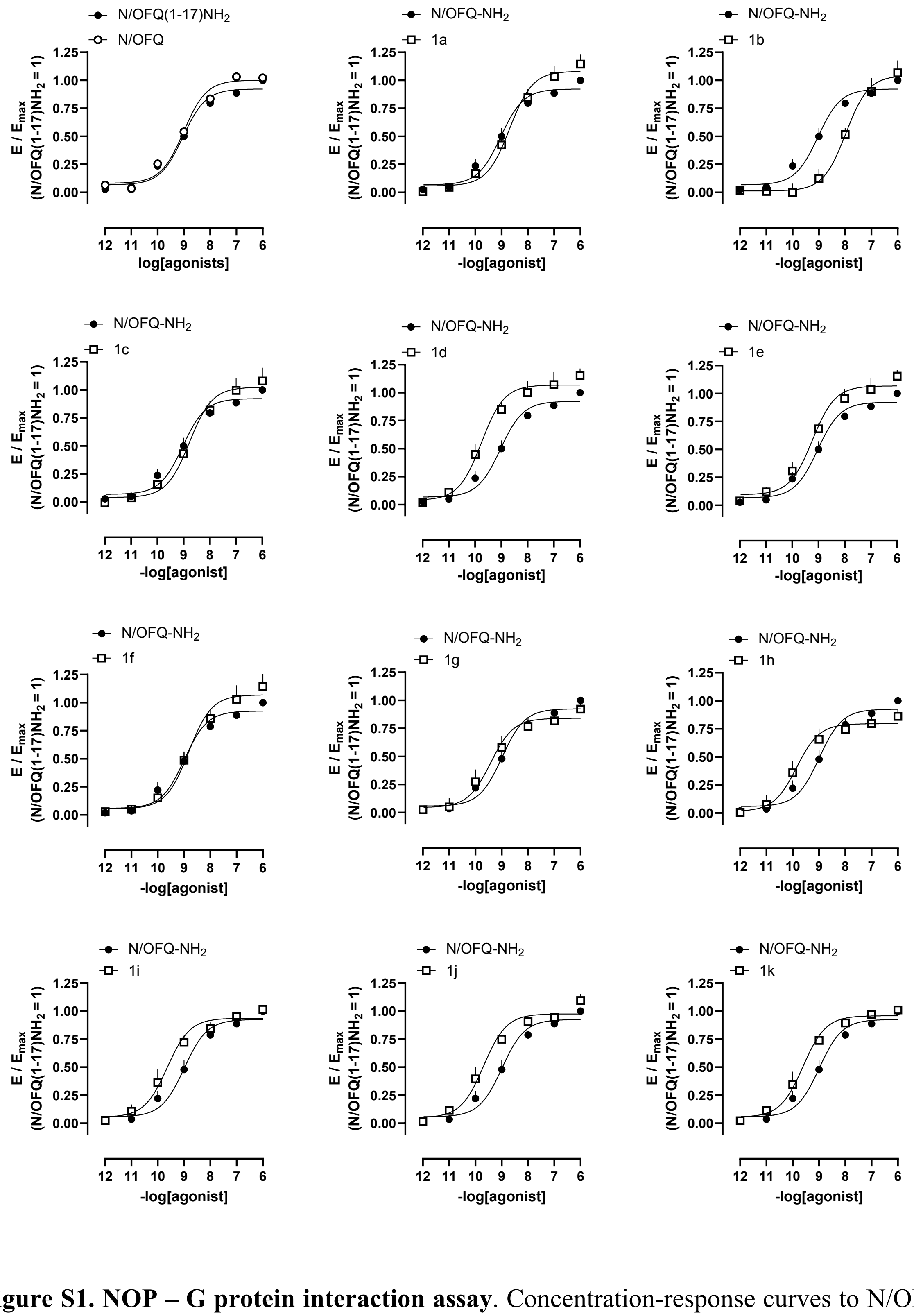

Figure S1. NOP – G protein interaction assay. Concentration-response curves to N/OFQ-NH_2 and dimeric derivatives in CHO cells stably expressing the NOP receptors. Data are the mean and sem of 5 experiments conducted in duplicate.

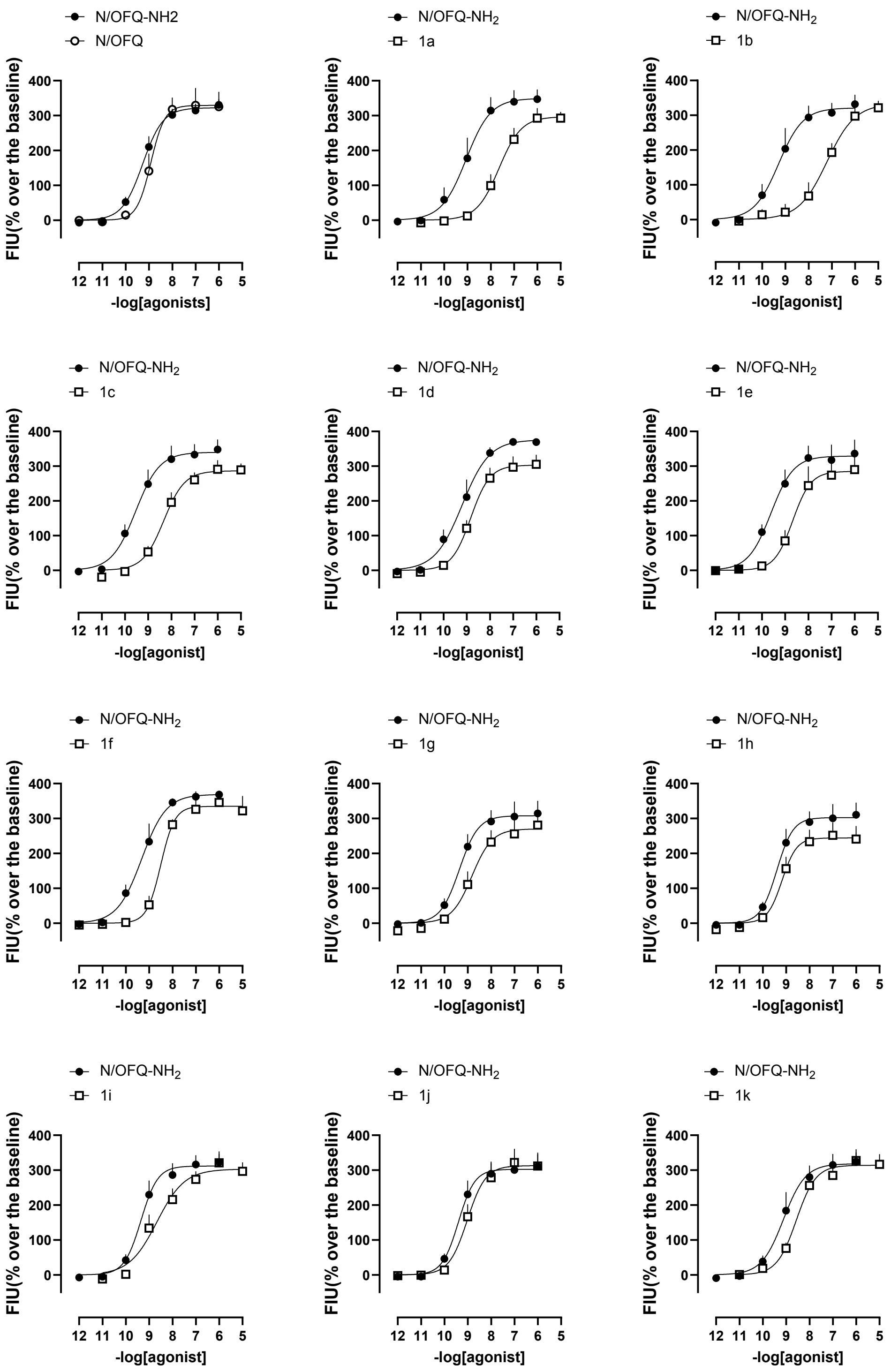

Figure S2. Calcium mobilization assay. Concentration-response curves to N/OFQ-NH$_2$ and dimeric derivatives CHO cells stably expressing the NOP receptors and chimeric Figure S2. Calcium mobilization assay G proteins. Data are the mean and sem of 5 experiments conducted in duplicate.

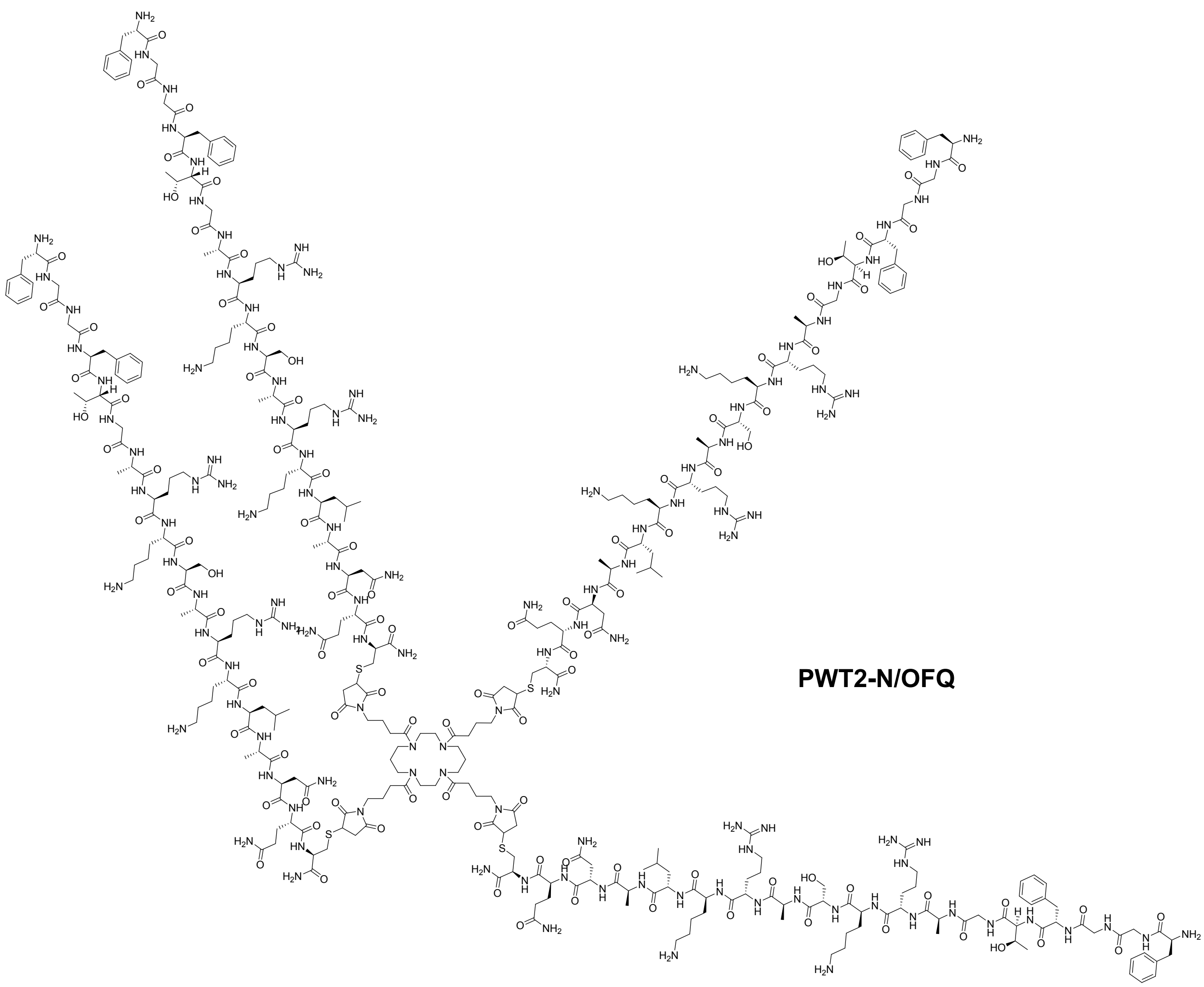

PWT2-N/OFQ

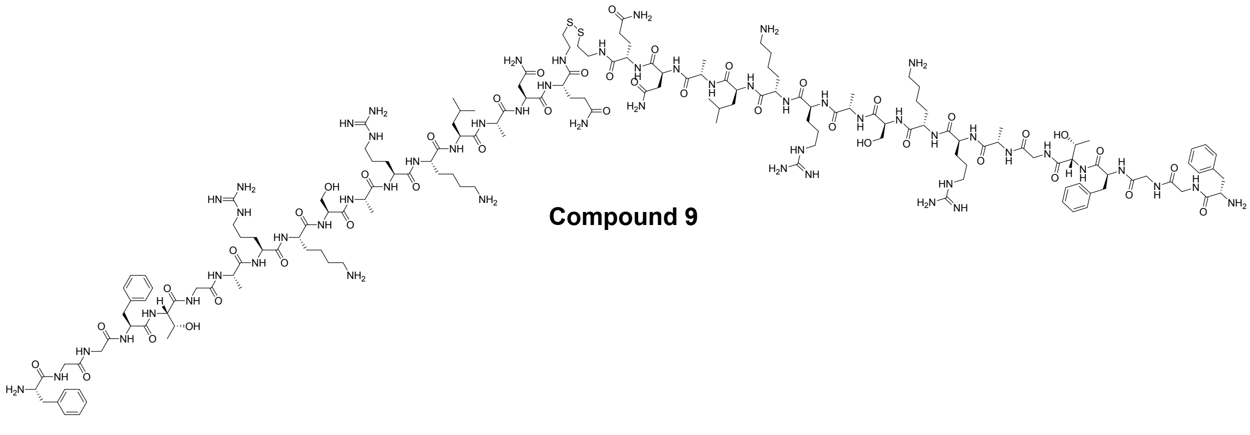

Compound 9

Figure S3. Chemical structure of compound 9 and PWT2-N/OFQ

HRMS Method

Compounds were dissolved in DMSO (200 µL, MS Grade) and a further dilution 1:100 was performed in H_2O:CH_3CN (MS Grade) 90:10 with 0.1% Formic acid (FA). 4uL of each solution were injected into a Vanquish Flex UHPLC coupled to an Orbitrap Exploris 120 HRMS (ALIFAR, UniPR). Compounds were separated on an ACQUITY UPLC BEH C18 (2.1x100mm, 1.7um ID) column, thermostated at 25°C, with a 6 min gradient of 0.1% FA in CH_3CN 2 to 80% vs 0.1% FA in water at 0.4mL/min. MS was operating in FullMS, 120000 resolving power. Scan parameters were set as follows: acquisition window 400-3000 m/z, ACG target: automatic, MaxIT 1000s, in positive ionization mode with an H-ESI source (positive voltage 3500V, sheath gas 40AU, capillary temperature 325°C, vaporizer 350°C). The instrument was calibrated with Vendor's calibration solution. Exact masses were extracted with FreeStyle v1.8 (Thermo Fisher). FullMS from TIC and isotopic abundances are reported in the present report. Monoisotopic derived masses for multicharged species were obtained with ThermoFisher Bayesian deconvolution algorithm.

Results

Adduct	Calculated monoisotopic m/z	Derived monoisotopic mass	Experimental m/z	Fit %
Found adducts with H^+	Monoisotopic mass of each z calculated from experimental m/z through Bayesian deconvolution algorithm	Total monoisotopic mass of the analyzed compound calculated from experimental m/z through Bayesian deconvolution algorithm	Measured m/z for that pattern	Dmass shift calculated vs experimental *100

HRMS and isotopic composition of dimeric peptides 1a-k

Compound 1a

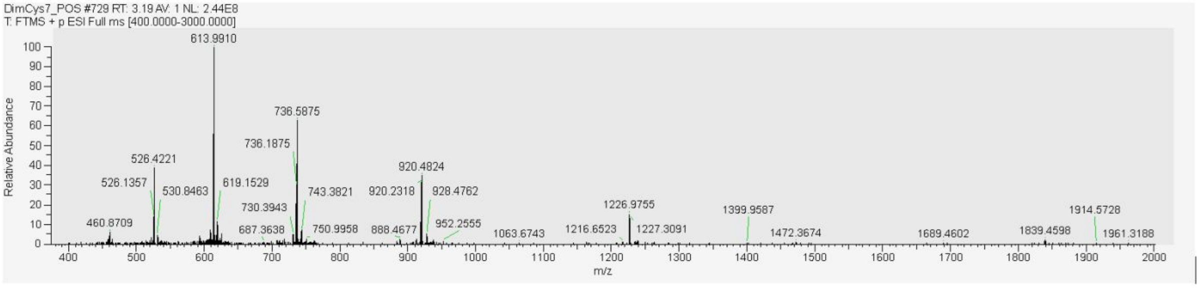

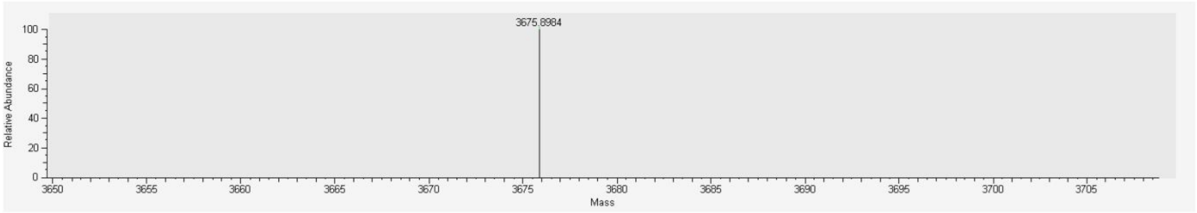

adduct	Calculated Monoisotopic m/z	Derived Monoisotopic Mass	Experimental m/z	Fit %
$[M+8H]^{8+}$	460.49452	3675.9008	460.74539	92.4
$[M+7H]^{7+}$	526.13555	3675.8991	526.42224	97.6
$[M+6H]^{6+}$	613.65693	3675.8973	613.99103	98.2
$[M+5H]^{5+}$	736.18686	3675.8974	736.58789	96.9
$[M+4H]^{4+}$	919.98175	3675.8957	920.4823	96.2
$[M+3H]^{3+}$	1226.30658	3675.9006	1226.97583	95
$[M+2H]^{2+}$	1838.95623	3675.8953	1839.95593	93

Theoretical m/z: 3675,8979

Observed m/z: 3675,8984

Error (ppm): 0.136021

Compound 1b

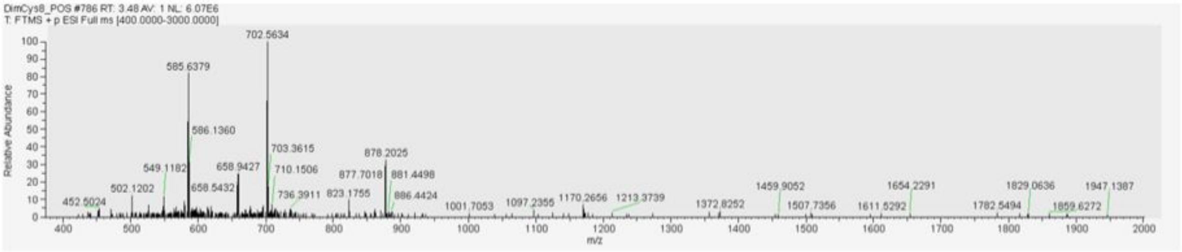

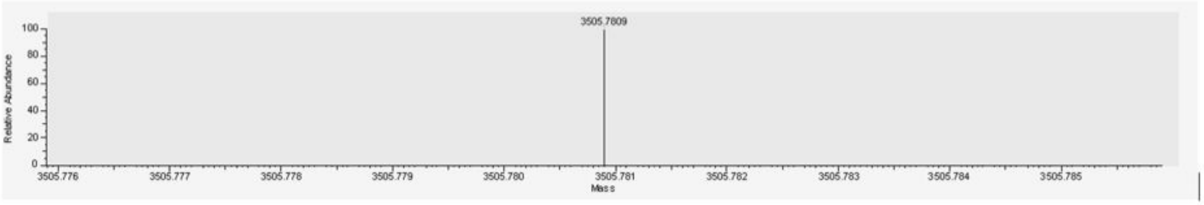

adduct	Calculated Monoisotopic m/z	Derived Monoisotopic Mass	Experimental m/z	Fit %
**[M+7H]$^{7+}$**	501.83243	3505.784	502.12018	94.4
**[M+6H]$^{6+}$**	585.30329	3505.7749	585.63788	95.5
**[M+5H]$^{5+}$**	702.1625	3505.7748	702.56342	94.2
**[M+4H]$^{4+}$**	877.4513	3505.7737	877.95209	93.9
**[M+3H]$^{3+}$**	1169.59931	3505.7781	1170.26562	96.6

Theoretical m/z: 3505.7761

Observed m/z: 3505.7809

Error (ppm): 1.369169

Compound 1c

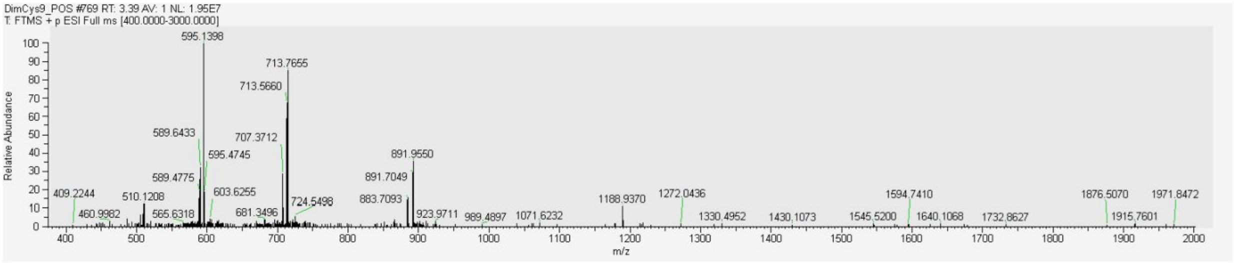

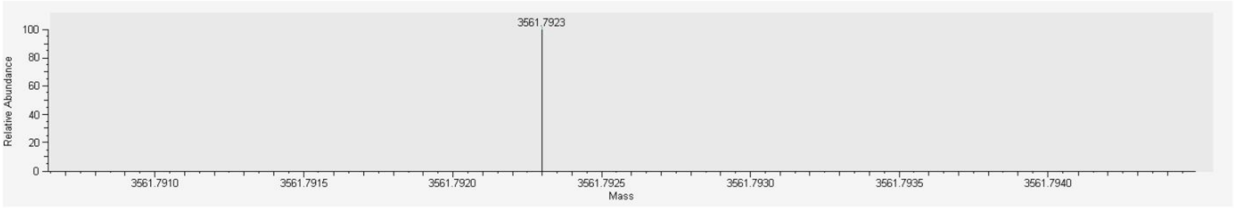

adduct	Calculated Monoisotopic m/z	Derived Monoisotopic Mass	Experimental m/z	Fit %
**[M+8H]$^{8+}$**	446.23085	3561.7934	446.48287	72
**[M+7H]$^{7+}$**	509.83421	3561.7903	510.12102	96
**[M+6H]$^{6+}$**	594.6387	3561.7897	594.97305	96.1
**[M+5H]$^{5+}$**	713.36499	3561.7884	713.76591	95.6
**[M+4H]$^{4+}$**	891.45442	3561.7862	891.95533	96.9
**[M+3H]$^{3+}$**	1188.27013	3561.7883	1188.93848	93.8

Theoretical m/z: 3561.7886

Observed m/z: 3561.7923

Error (ppm): 1.038804

Compound 1d

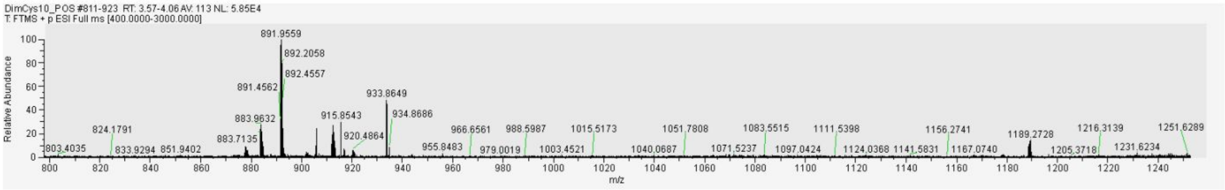

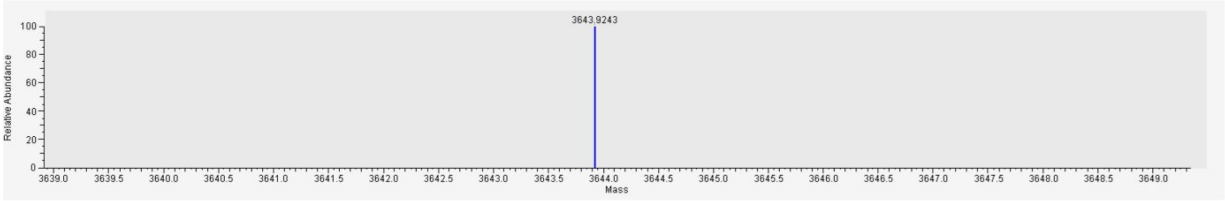

adduct	Calculated Monoisotopic m/z	Derived Monoisotopic Mass	Experimental m/z	Fit %
**[M+7H]$^{7+}$**	521.56686	3643.9174	521.85381	96.9
**[M+6H]$^{6+}$**	608.32679	3643.9187	608.66144	95.4
**[M+5H]$^{5+}$**	729.79069	3643.9155	730.19161	95.8
**[M+4H]$^{4+}$**	911.98655	3643.9149	912.48725	92.3

Theoretical m/z: 3643.9171

Observed m/z: 3643.9243

Error (ppm): 1.975896

Compound 1e

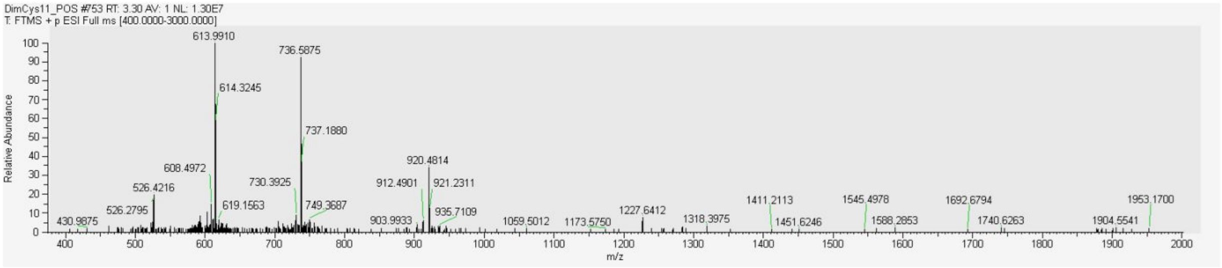

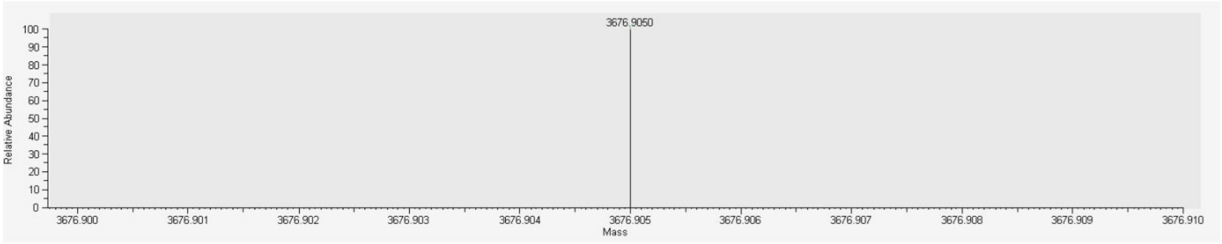

adduct	Calculated Monoisotopic m/z	Derived Monoisotopic Mass	Experimental m/z	Fit %
**$[M+7H]^{7+}$**	526.27774	3676.8957	526.56464	89.7
**$[M+6H]^{6+}$**	613.82282	3676.8956	614.15692	94.5
**$[M+5H]^{5+}$**	736.38593	3676.8985	736.78796	95.2
**$[M+4H]^{4+}$**	920.23059	3676.8945	920.73138	94.4
**$[M+3H]^{3+}$**	1226.63837	3676.9007	1227.31287	70.9

Theoretical m/z: 3676.8933

Observed m/z: 3676.9050

Error (ppm): 3.182034

Compound 1f

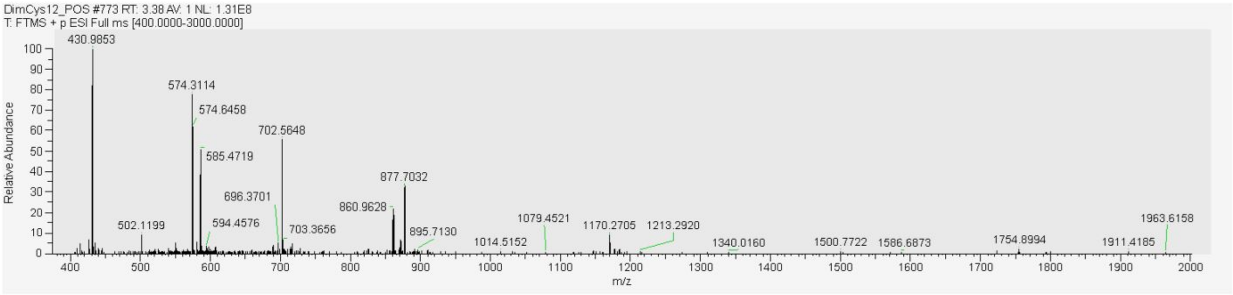

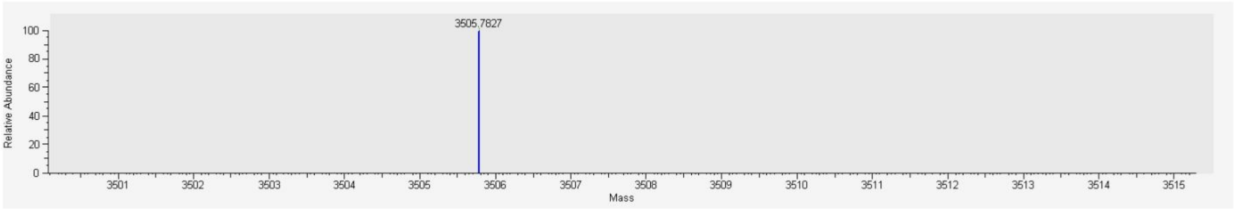

adduct	Calculated Monoisotopic m/z	Derived Monoisotopic Mass	Experimental m/z	Fit %
**[M+7H]$^{7+}$**	501.83339	3505.7843	502.11987	91.7
**[M+6H]$^{6+}$**	585.30441	3505.7824	585.63849	97.3
**[M+5H]$^{5+}$**	702.16384	3505.7827	702.56476	96.4
**[M+4H]$^{4+}$**	877.45298	3505.7789	877.95343	88.7
**[M+3H]$^{3+}$**	1169.60155	3505.7816	1170.27051	96

Theoretical m/z: 3505.7828

Observed m/z: 3505.7827

Error (ppm): -0.028524

Compound 1g

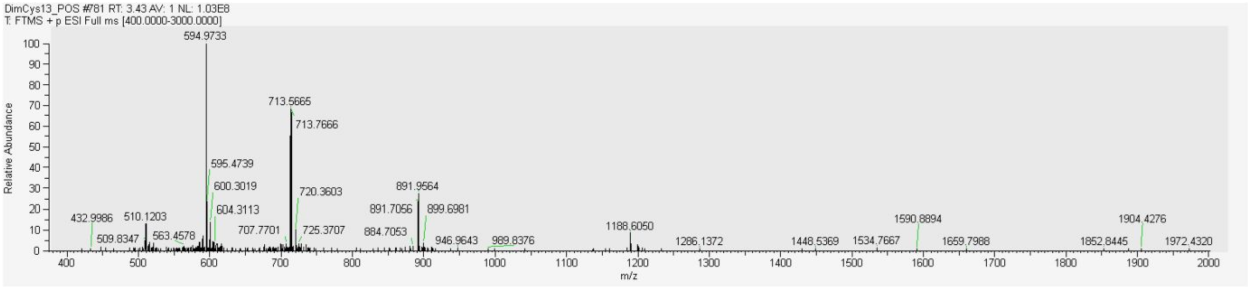

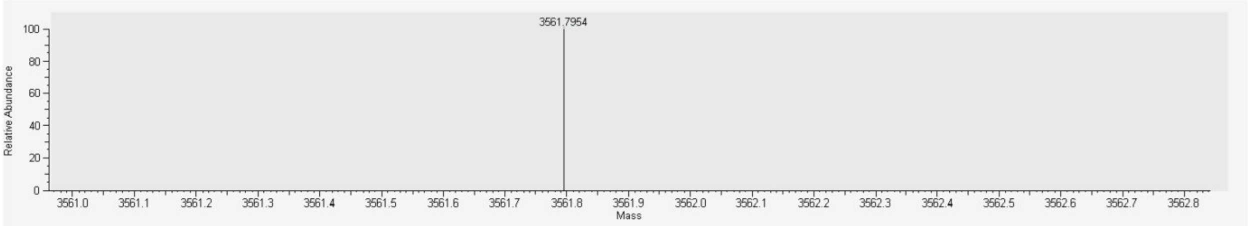

adduct	Calculated Monoisotopic m/z	Derived Monoisotopic Mass	Experimental m/z	Fit %
**$[M+7H]^{7+}$**	509.83461	3561.7909	510.12033	92
**$[M+6H]^{6+}$**	594.63917	3561.7925	594.97327	95.7
**$[M+5H]^{5+}$**	713.36554	3561.7912	713.7666	97.6
**$[M+4H]^{4+}$**	891.45511	3561.7889	891.95636	96.9
**$[M+3H]^{3+}$**	1188.27106	3561.7927	1188.94055	82.5

Theoretical m/z: 3561.7913

Observed m/z: 3561.7954

Error (ppm): 1.151106

Compound 1h

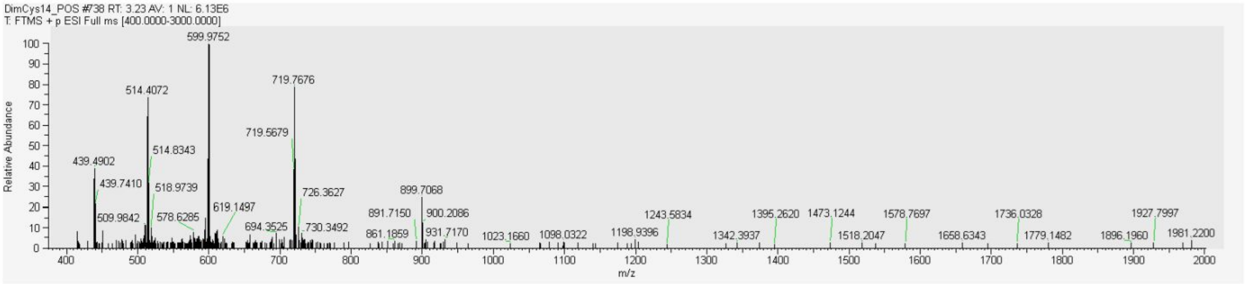

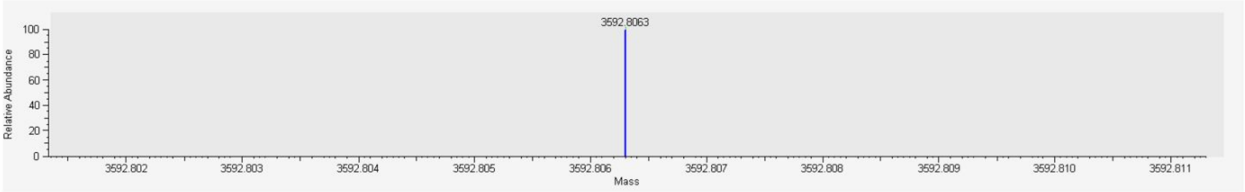

adduct	Calculated Monoisotopic m/z	Derived Monoisotopic Mass	Experimental m/z	Fit %
**[M+7H]$^{7+}$**	514.26383	3592.8015	514.55157	84.4
**[M+6H]$^{6+}$**	599.80659	3592.7964	600.14069	95.5
**[M+5H]$^{5+}$**	719.56645	3592.796	719.96741	95.5
**[M+4H]$^{4+}$**	899.20624	3592.7942	899.70679	87.1

Theoretical m/z: 3592,7959

Observed m/z: 3592,8063

Error (ppm): 2.894682

Compound 1i

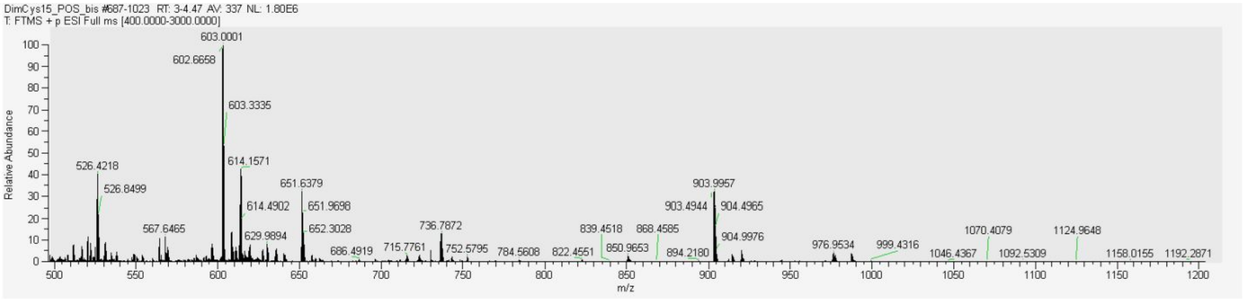

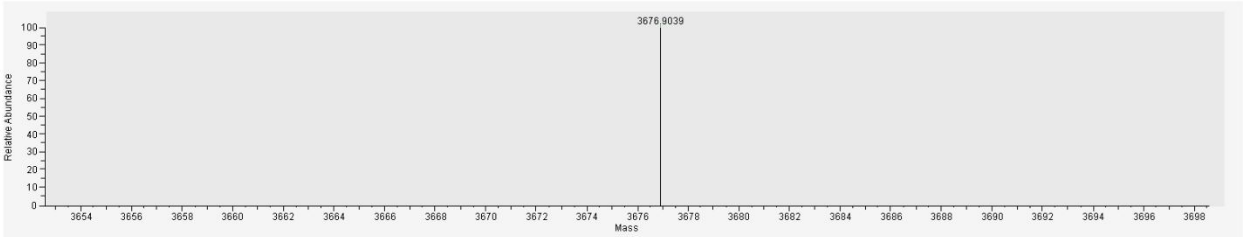

adduct	Calculated Monoisotopic m/z	Derived Monoisotopic Mass	Experimental m/z	Fit %
**[M+7H]$^{7+}$**	526.27791	3676.8936	526.56422	97.4
**[M+6H]$^{6+}$**	613.82301	3676.8941	614.15711	98
**[M+5H]$^{5+}$**	736.38616	3676.8943	736.78719	97.6
**[M+4H]$^{4+}$**	920.23088	3676.9023	920.73222	86.1

Theoretical m/z: 3676.8944

Observed m/z: 3676.9039

Error (ppm): 2.583702

Compound 1j

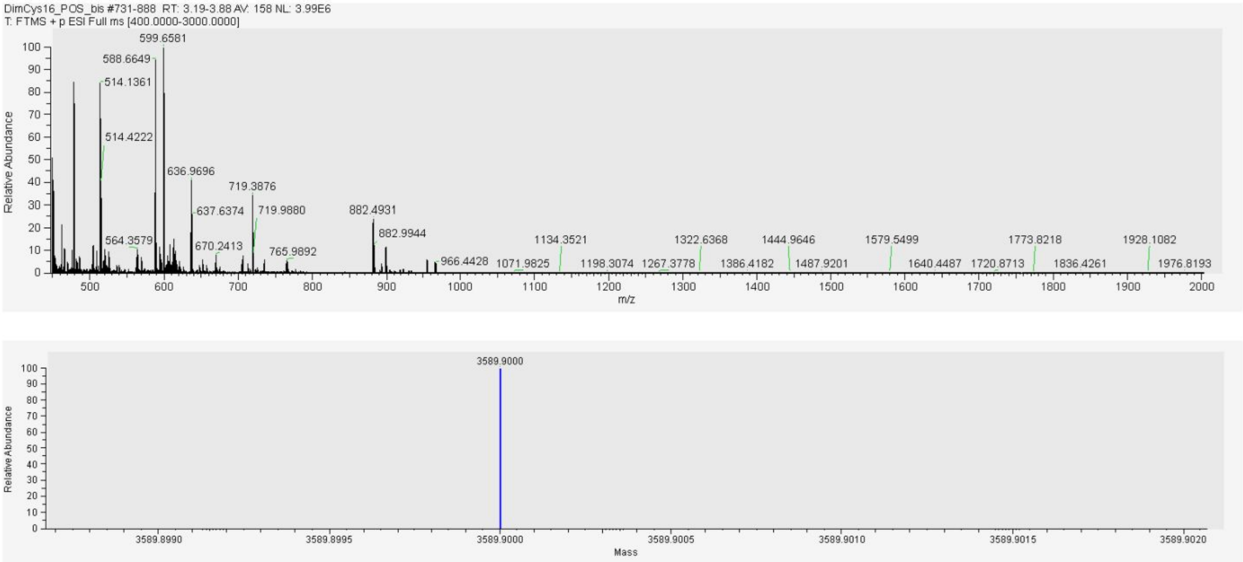

adduct	Calculated Monoisotopic m/z	Derived Monoisotopic Mass	Experimental m/z	Fit %
**$[M+8H]^{8+}$**	449.74478	3589.9016	449.99636	81.2
**$[M+7H]^{7+}$**	513.85014	3589.8968	514.13613	98.1
**$[M+6H]^{6+}$**	599.32395	3589.8978	599.65805	96.2
**$[M+5H]^{5+}$**	718.98729	3589.8963	719.38764	96.4
**$[M+4H]^{4+}$**	898.48229	3589.8984	898.98367	92.8

Theoretical m/z: 3589.900048

Observed m/z: 3589.9000

Error (ppm): -0.013371

Compound 1k

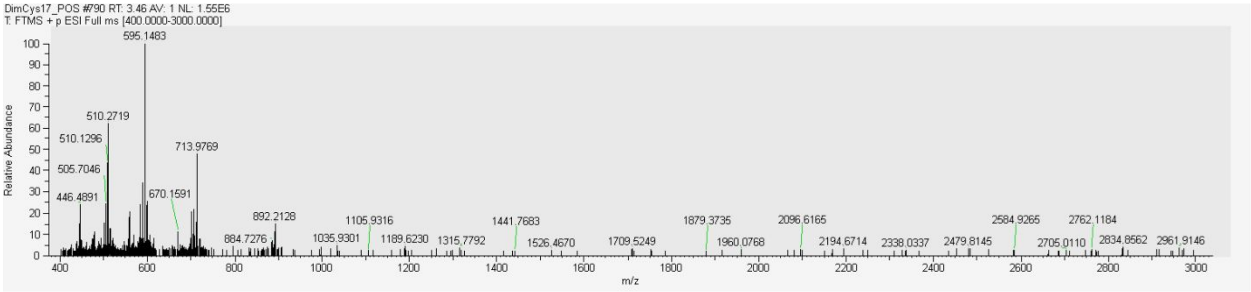

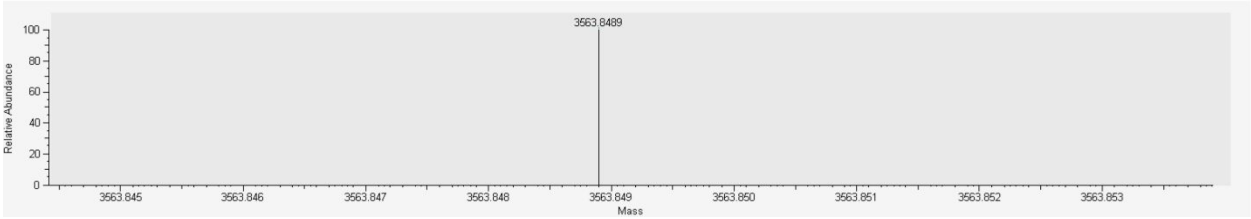

adduct	Calculated Monoisotopic m/z	Derived Monoisotopic Mass	Experimental m/z	Fit %
$[M+7H]^{7+}$	510.12779	3563.8445	510.4137	92
$[M+6H]^{6+}$	594.9812	3563.8381	595.31464	95.7
$[M+5H]^{5+}$	713.77599	3563.8365	714.1748	93.4

Theoretical m/z: 3563.8435

Observed m/z: 3563.8489

Error (ppm): 1.515218

HPLC traces of the final dimeric peptides 1a-k

Compound 1a

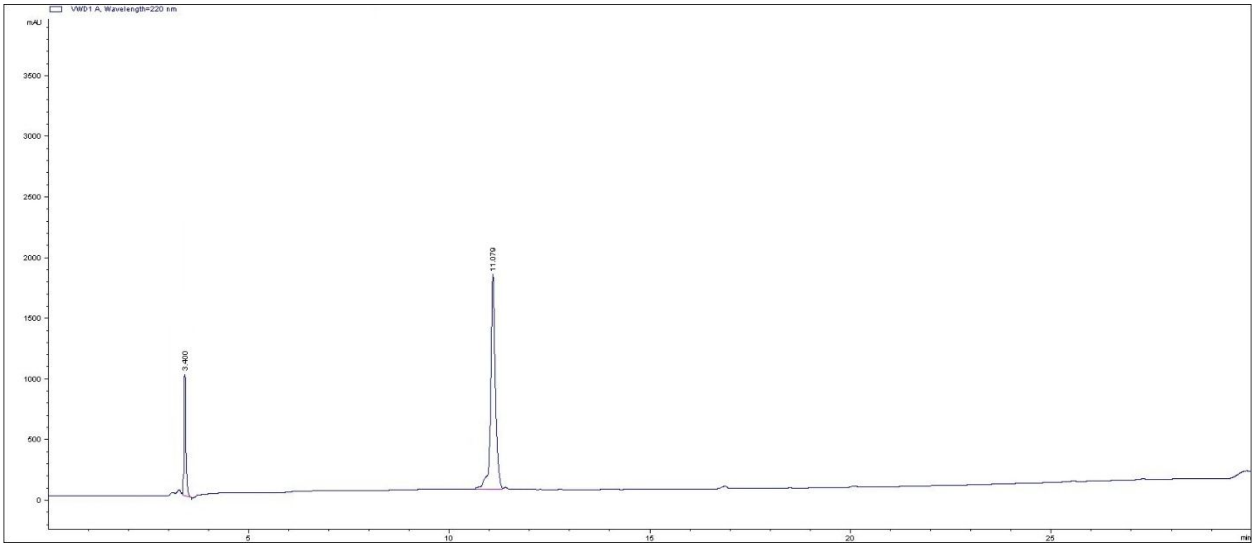

Compound 1b

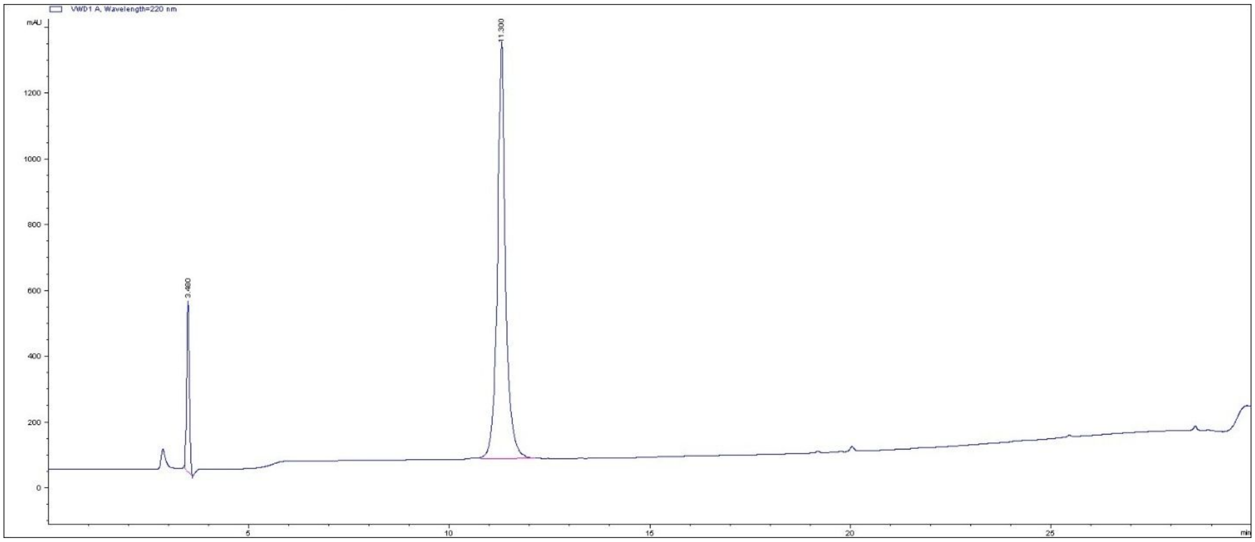

Compound 1c

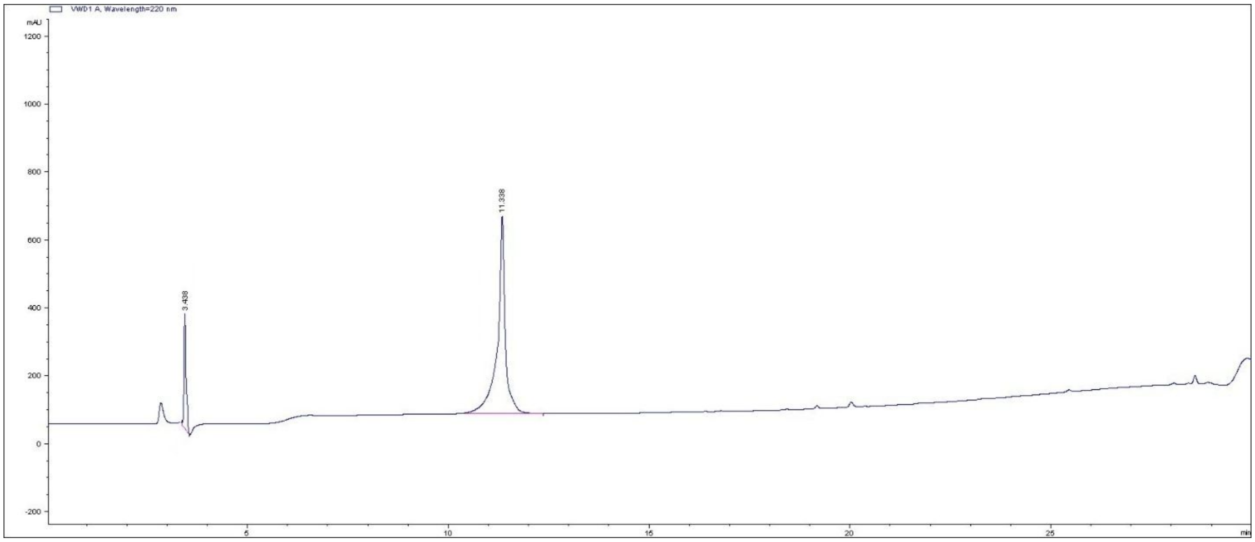

Compound 1d

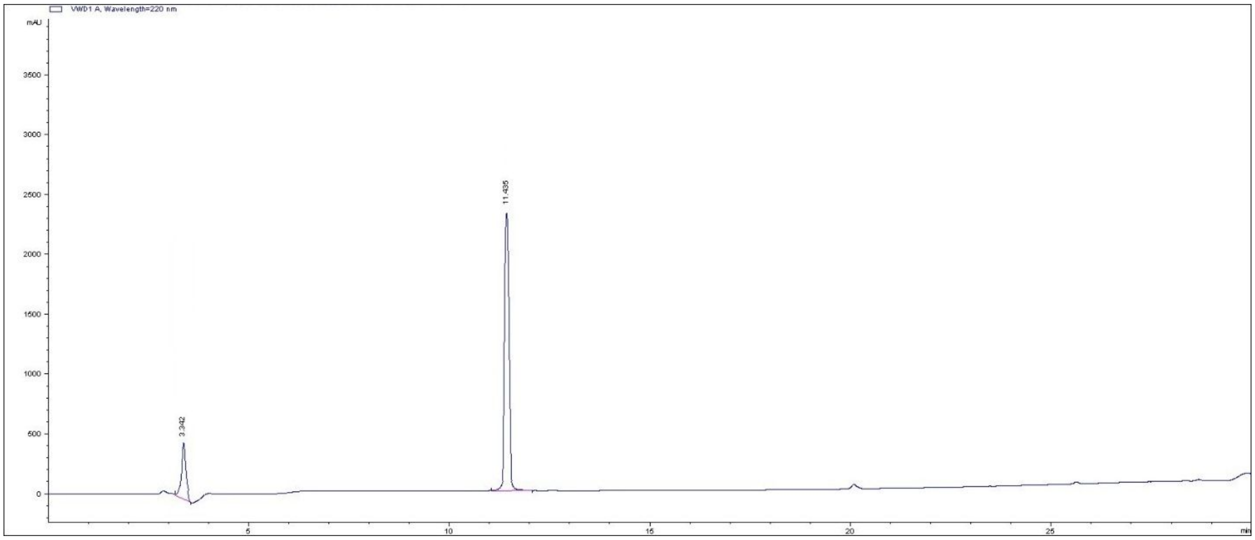

Compound 1e

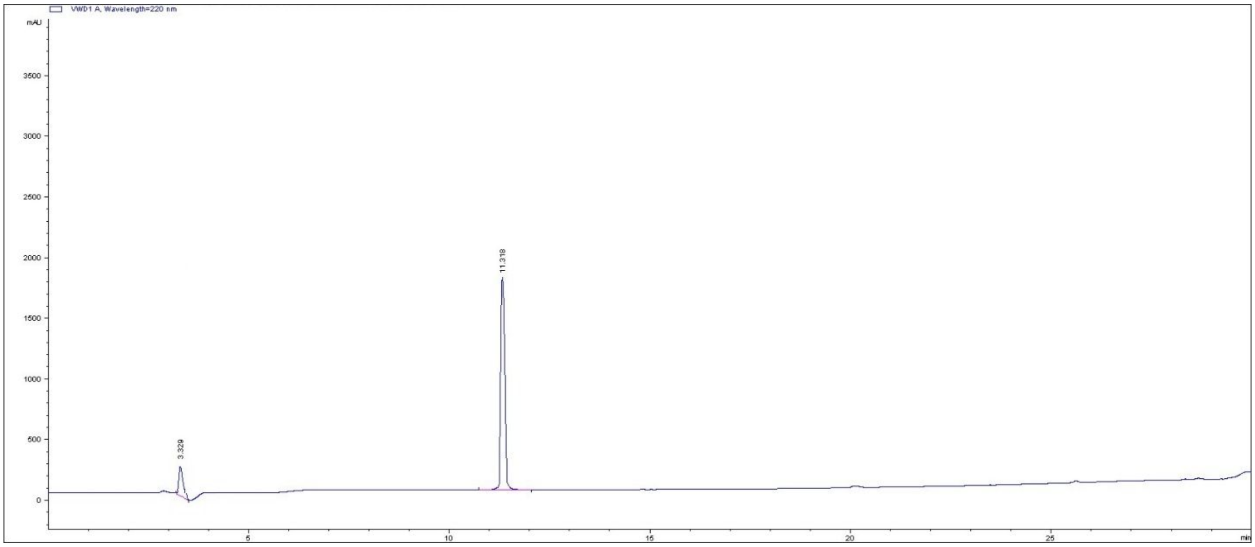

Compound 1f

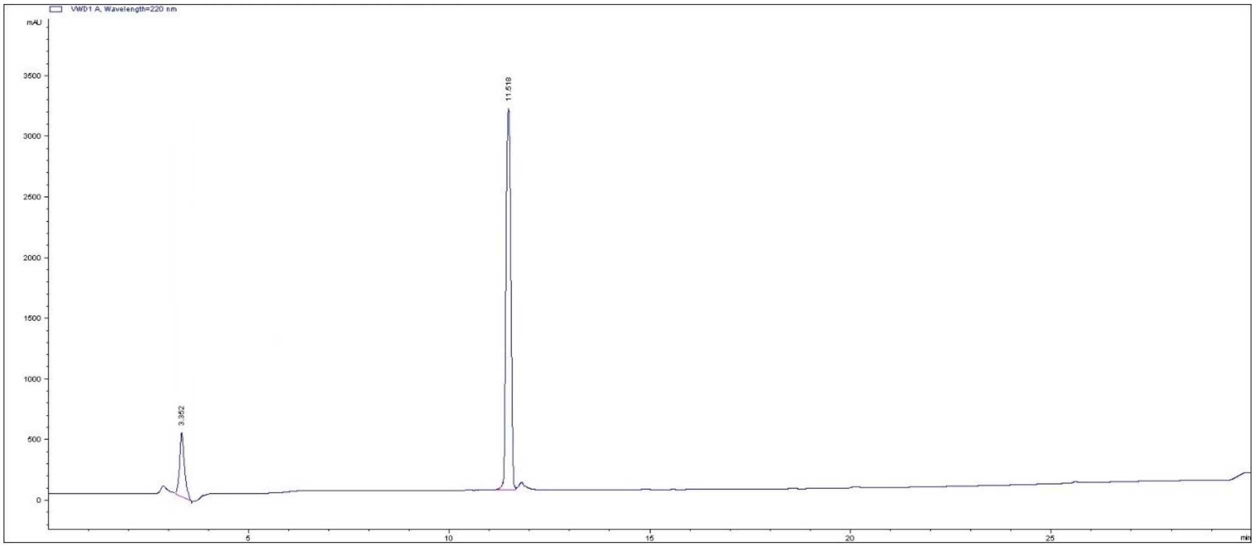

Compound 1g

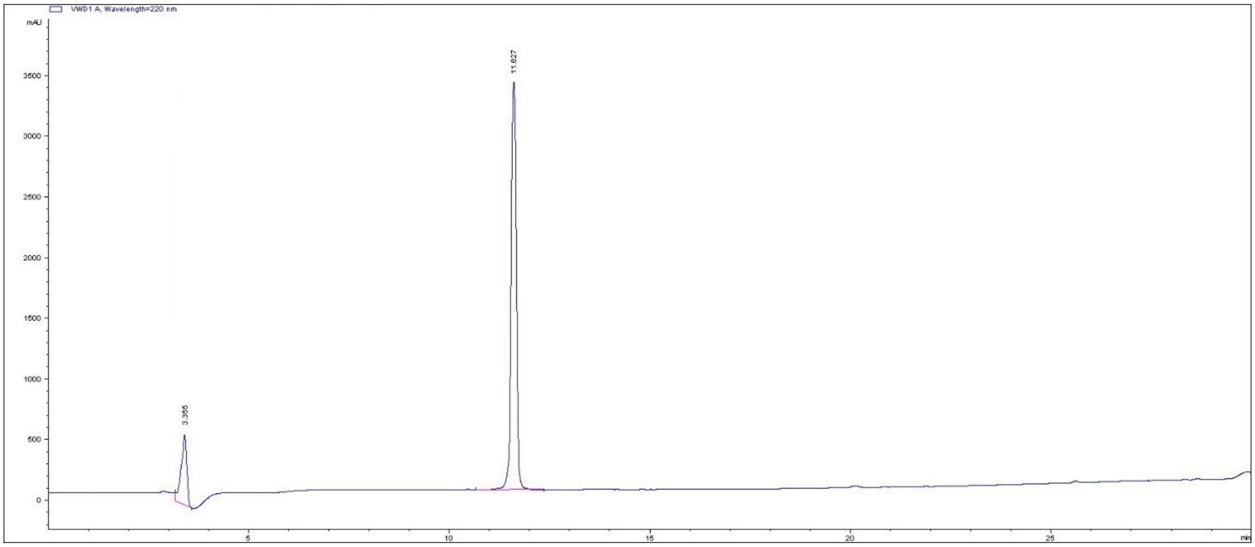

Compound 1h

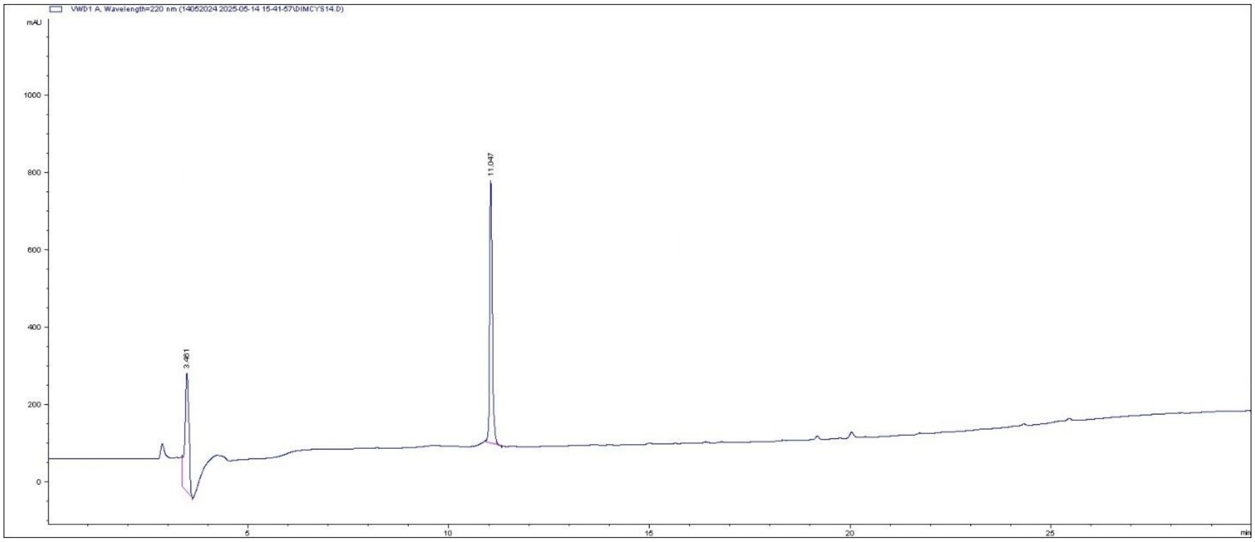

Compound 1i

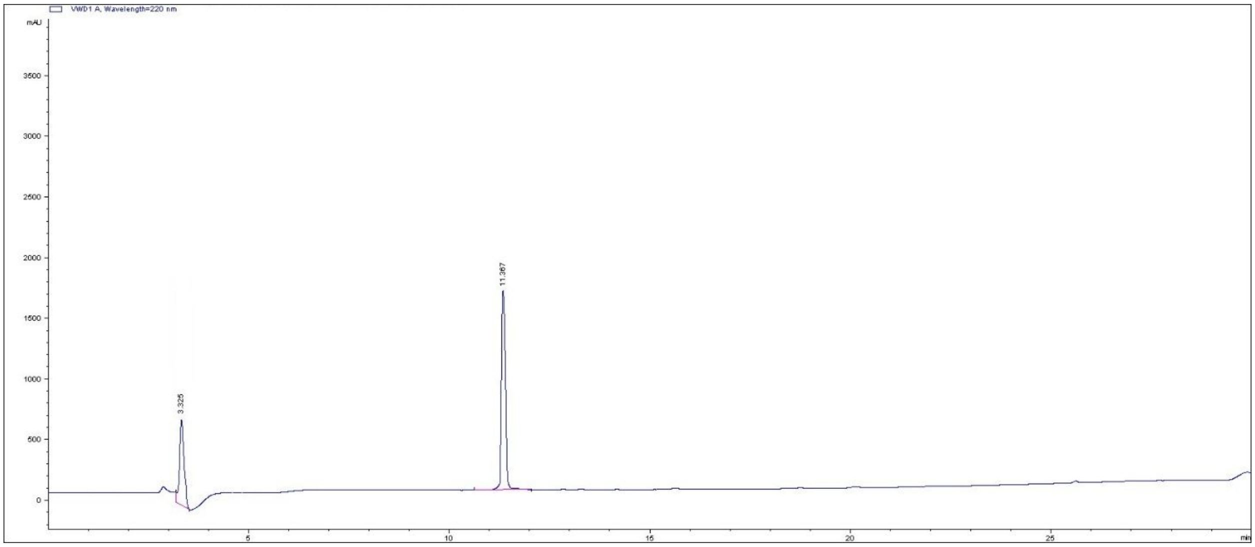

Compound 1j

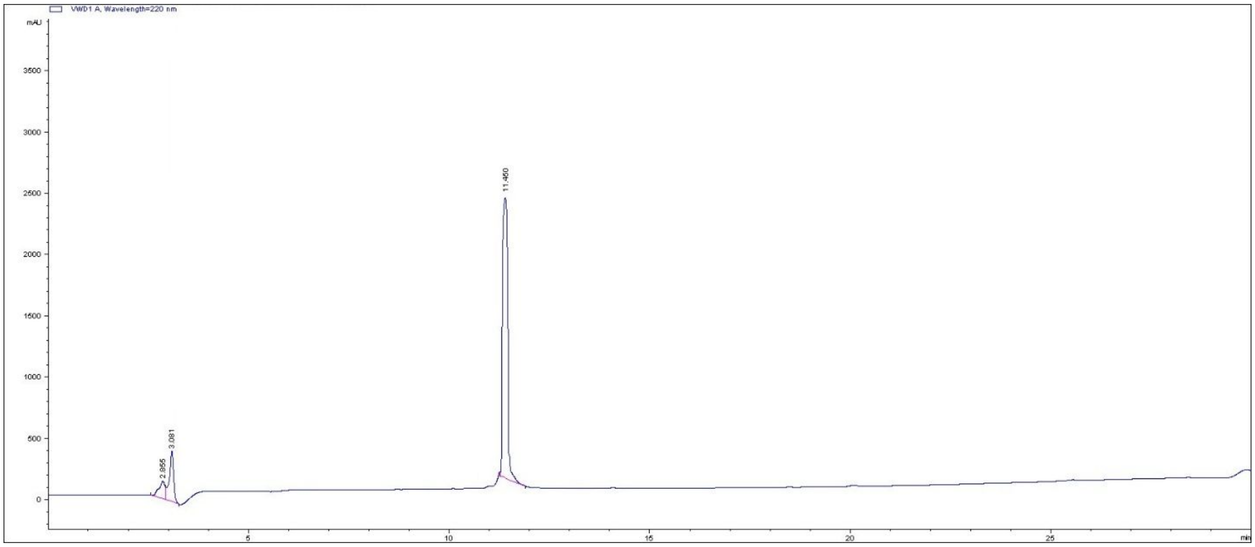

Compound 1k

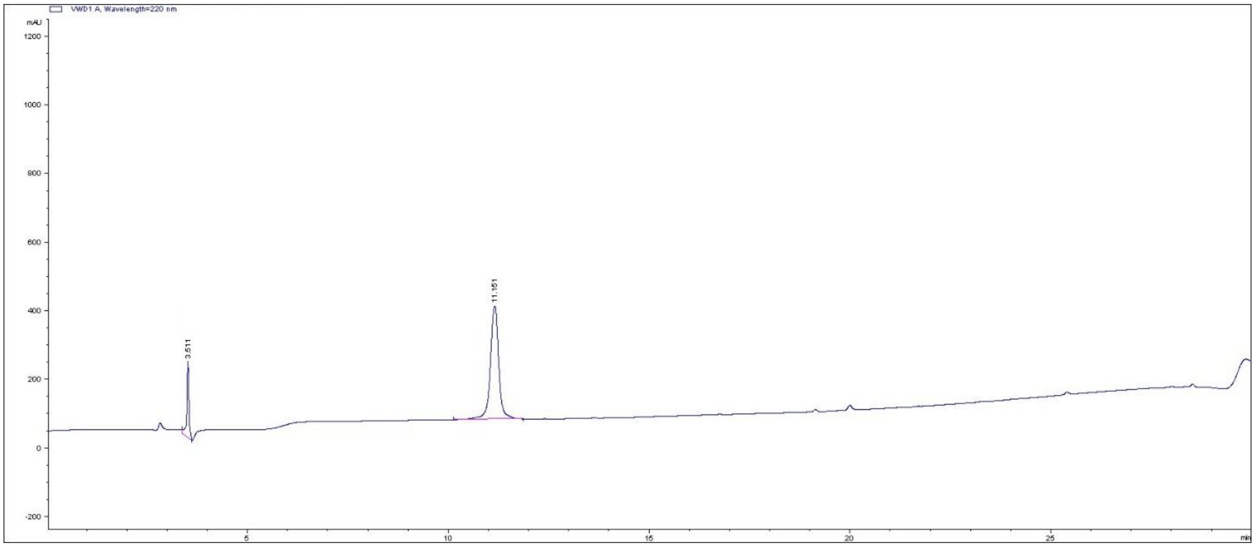

VWD1 A, Wavelength=220 nm
mAU
1200
1000
800
600
400
200
0
-200
3.511
11.151
5
10
15
20
25
min
